# Two-dimensional artificial light-harvesting antennae with predesigned high-order structure and robust photosensitising activity

**DOI:** 10.1038/srep32944

**Published:** 2016-09-13

**Authors:** Xiao Feng, Xuesong Ding, Long Chen, Yang Wu, Lili Liu, Matthew Addicoat, Stephan Irle, Yuping Dong, Donglin Jiang

**Affiliations:** 1 Field of Environment and Energy, School of Materials Science, Japan Advanced Institute of Science and Technology, 1-1 Asahidai, Nomi 923-1292, Japan; 2 College of Materials Science and Engineering, Beijing Institute of Technology, Zhongguancun South Street, Beijing, 100081, China; 3 WPI-Research Initiative-Institute of Transformative Bio-Molecules and Department of Chemistry, Graduate School of Science, Nagoya University, Furo-cho, Chikusa-ku, Nagoya 464-8602, Japan

## Abstract

Highly ordered discrete assemblies of chlorophylls that are found in natural light-harvesting antennae are key to photosynthesis, which converts light energy to chemical energy and is the principal producer of organic matter on Earth. Porphyrins and phthalocyanines, which are analogues of chlorophylls, exhibit a strong absorbance of visible and near-infrared light, respectively. A highly ordered porphyrin-co-phthalocyanine antennae would harvest photons over the entire solar spectrum for chemical transformation. However, such a robust antennae has not yet been synthesised. Herein, we report a strategy that merges covalent bonds and noncovalent forces to produce highly ordered two-dimensional porphyrin-co-phthalocyanine antennae. This methodology enables control over the stoichiometry and order of the porphyrin and phthalocyanine units; more importantly, this approach is compatible with various metalloporphyrin and metallophthalocyanine derivatives and thus may lead to the generation of a broad structural diversity of two-dimensional artificial antennae. These ordered porphyrin-co-phthalocyanine two-dimensional antennae exhibit unique optical properties and catalytic functions that are not available with single-component or non-structured materials. These 2D artificial antennae exhibit exceptional light-harvesting capacity over the entire solar spectrum as a result of a synergistic light-absorption effect. In addition, they exhibit outstanding photosensitising activities in using both visible and near-infrared photons for producing singlet oxygen.

Highly efficient, robust artificial light-harvesting antennae are essential for the collection, capture, and conversion of solar energy, which is a major renewable green-energy resource. The solar energy that reaches Earth is distributed predominately over the visible and near-infrared regions at a low energy density. The highly ordered structure of chlorophylls found in light-harvesting antenna complexes in plants and bacteria^1^,^2^ has inspired the synthesis of highly ordered antenna structures of synthetic pigments for artificial light harvesting. Porphyrins and phthalocyanines, which are artificial analogues of chlorophylls, enable extended π-cloud delocalisation over their multiple-electron π-systems^3^. These analogues can coordinate with nearly all transition metals to form various metalloporphyrins and metallophthalocyanines, and they exhibit a broad diversity of remarkable functions, including optical, photochemical, electrical, magnetic, catalytic, and redox properties^3^,^4^,^5^. In addition, porphyrins and phthalocyanines are complementary in light absorption because porphyrins absorb strongly in the visible region and phthalocyanines exhibit powerful absorbance in the near-infrared region. Therefore, if these two absorption features can be merged, the resulting materials would cover the entire visible and near-infrared regions. The production of ordered porphyrin-*co*-phthalocyanine antennae has been a long-standing challenge. However, robust materials have not yet been achieved by using either covalent or noncovalent approaches^5^.

Herein, we report a strategy that merges covalent bonding with noncovalent interactions to produce highly ordered porphyrin-*co*-phthalocyanine two-dimensional (2D) light-harvesting antennae. The covalent bonding links porphyrins and phthalocyanines via topology-directed polycondensation into extended 2D sheets that crystallise via noncovalent interactions to form 2D covalent organic frameworks (COFs) with periodic porphyrin and phthalocyanine segregated columns and unidirectional open nanochannels. COFs have been reported for the construction of various π-systems^6^,^7^,^8^,^9^,^10^,^11^,^12^,^13^,^14^,^15^,^16^,^17^,^18^,^19^,^20^,^21^,^22^,^23^,^24^,^25^,^26^,^27^,^28^,^29^,^30^,^31^,^32^,^33^,^34^,^35^, and porphyrins and phthalocyanines have been explored for the preparation of 2D COFs^14^,^17^,^18^,^19^,^21^,^22^,^23^,^24^,^25^,^27^,^30^, which enable highly ordered structures of porphyrins or phthalocyanines, respectively. In this study, the topological design, controlled synthesis, and precise tune of highly ordered porphyrin-*co*-phthalocyanine structures are reported. Our strategy not only dictates the primary sheet structure but also controls the high-order framework structure, creating unique and periodically aligned π-columns and unidirectional 1D channel arrays. The successful structural ordering allows for the construction of robust artificial light-harvesting antennae. The 2D porphyrin-*co*-phthalocyanine antennae harvest photons with a wide range of energies and exhibit robust photosensitising activities in using solar energy for activation of molecular oxygen. To generate singlet oxygen, these COFs serve as antennae to harvest light and produce the triplet state, from which the excitation energy is transfer to molecular oxygen to generate singlet oxygen. Therefore, the energy transfer process involved in these COFs is a triplet-to-triplet excited state energy transfer. By contrast, the energy transfer involved in the nature light-harvesting antennae is a singlet-to-singlet excited state energy transfer process.

## Results

### Topological and molecular designs

**Figure 1:**
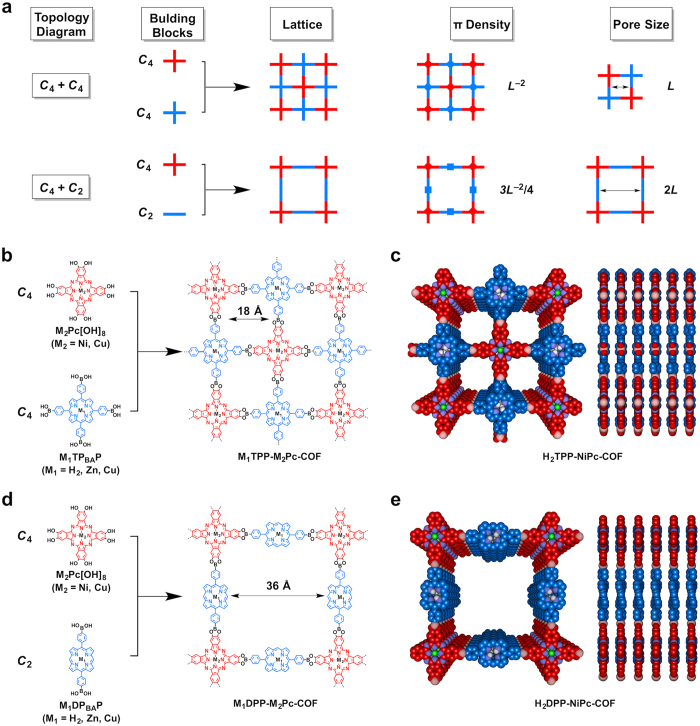
Topology diagrams and synthesis of highly ordered 2D porphyrin-*co*-phthalocyanine antennae. (**a**) The *C*_4_ + *C*_4_ and *C*_4_ + *C*_2_ topology diagrams for the design of porphyrin-*co*-phthalocyanine COFs and their difference in the lattice, π-density and pore size. (**b**) Schematic of the synthesis of *C*_4_ + *C*_4_-diagram-based M_1_TPP-M_2_Pc-COFs. (**c**) Top and side views of 2D H_2_TPP-NiPc-COF. (**d**) Schematic of the synthesis of *C*_4_ + *C*_2_-diagram-based M_1_DPP-M_2_Pc-COFs. (**e**) Top and side views of 2D H_2_DPP-NiPc-COF.

We and other groups have explored a *C*_4_ + *C*_2_ topological diagram for the synthesis of porphyrin or phthalocyanine COFs^14^,^17^,^18^,^19^,^21^,^22^,^23^,^24^,^25^,^27^,^30^. The *C*_4_ + *C*_2_ diagram leads to the generation of mesoporous porphyrin or phthalocyanine COFs, because of the large size of the porphyrin or phthalocyanine units. We developed a new topological diagram based on the *C*_4_ + *C*_4_ combination (Fig. 1a). For the first time, this new *C*_4_ + *C*_4_ topological diagram enables the direct synthesis of microporous porphyrin-*co*-phthalocyanine COFs. More importantly, this *C*_4_ + *C*_4_ diagram significantly enhances the density of π-columns (*L*^−2^) to 1.33 fold that of the *C*_4_ + *C*_2_ topology (3/4*L*^−2^). The *C*_4_ + *C*_4_ diagram substantially decreases the pore size (*L*) to only half that of the *C*_4_ + *C*_2_ topology (2*L*). Both diagrams enable the incorporation of porphyrin and phthalocyanine units into highly ordered 2D structures, and the different diagrams lead to a broad diversity of highly ordered 2D light-harvesting antennae.

### *C*_4_ + *C*_4_ diagram and microporous porphyrin-*co*-phthalocyanine 2D antennae

We designed a *C*_4_ freebase tetraphenylporphyrin with four boronic acid groups (H_2_TP_BA_P) and a *C*_4_ nickel phthalocyanine with eight hydroxyl units (NiPc[OH]_8_) as monomers for the polycondensation reaction (Fig. 1b). The reaction between the boronic acid and diol is an authentic condensation that leads to the formation of a planar boronate ester ring, which contributes to the formation of a planar 2D structure. Because the boronic acid and diol reactive groups on the two monomers are oriented in a *C*_4_ geometry, the *C*_4_ + *C*_4_ diagram guides the formation of tetragonal sheets with both units located at the vertices (Fig. 1b, M_1_TPP-M_2_Pc-COF; M_1_ = H_2_, M_2_ = Ni). The polycondensation reaction of H_2_TP_BA_P and NiPc[OH]_8_ loaded in a stoichiometric equimolar ratio was conducted in DMAc/*o*-DCB (1/1 v/v) at 120 °C over 7 days under solvothermal conditions to produce a black solid in 76% isolated yield. Infrared spectroscopy (IR) revealed the formation of boronate ester linkages between the two π-macrocycles (Supplementary Fig. 1). Field-emission scanning electron microscopy (FE-SEM) indicated that the H_2_TPP-NiPc-COF assumed a disk-like morphology with a size on the micrometer scale (Supplementary Fig. 2). High-resolution transmission electron microscopy (HR-TEM) revealed parallel aligned and straight extended lines (Supplementary Fig. 3). Notably, the π-grid lines were evenly aligned with a distance of 1.8 nm, which is consistent with the theoretical micropore size (1.8 nm).

### Crystal structure

**Figure 2:**
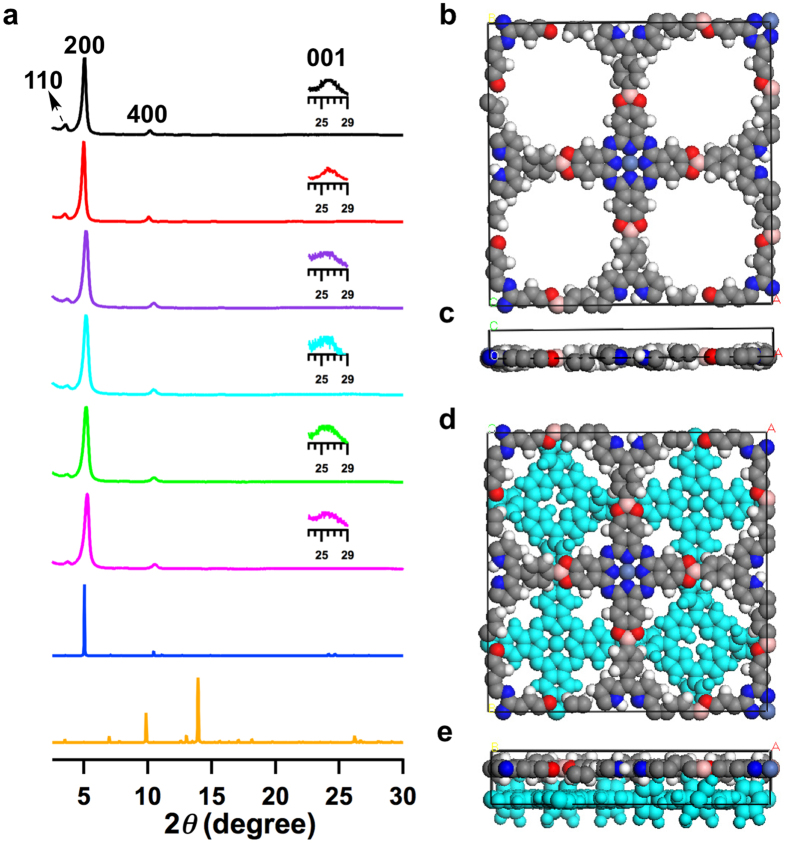
Crystalline structures of microporous 2D porphyrin-co-phthalocyanine COFs. (**a**) Experimentally observed XRD patterns (black curve for H_2_TPP-NiPc-COF, red curve for H_2_DPP-CuPc-COF, purple curve for ZnTPP-NiPc-COF, sky-blue curve for ZnTPP-CuPc-COF, green curve for CuTPP-NiPc-COF and magenta curve for CuTPP-CuPc-COF) and patterns simulated with the eclipsed AA stacking (blue curve) for H_2_TPP-NiPc-COF and with the AB-staggered stacking (orange curve) for H_2_TPP-NiPc-COF. (**b**,**c**) Views of the unit cell along the *z* (**b**) and *y* (**c**) axes of H_2_TPP-NiPc-COF. (**d**,**e**) Views of the AB-staggered unit cell along the *z* (**d**) and *y* (**e**) axes. Insets in XRD profiles show the enlarged views of the (001) peaks.

The 2D materials exhibit very intense X-ray diffraction (XRD) peaks (Fig. 2a, red curve). The peak at 2*θ* = 4.9° corresponds to the (200) facet of a periodically ordered 2D tetragonal lattice, and the peaks at 2*θ* angles of 3.5° and 9.9° are attributed to the (110) and (400) facets, respectively. The regularity along the stacking direction is confirmed by the peak at approximately 26° (Fig. 2a, Inset), yielding an interlayer distance of 3.5 Å. Therefore, the *C*_4_ + *C*_4_ diagram enables the direct synthesis of highly ordered porphyrin-*co*-phthalocyanine 2D materials upon geometry-directed polycondensation.

Structural reconstructions were achieved from the density-functional-based tight-binding (DFTB) method and provided an XRD pattern (Fig. 2a, blue curve) for an eclipsed AA stacking mode that agreed well with the experimentally observed profile (black curve). Figure 2b,c show the top and side views of a unit cell, respectively. The AA stacking structure assumes the superposition of sheets with crystalline parameters of *a* = *b* = 35.90 Å. In contrast, the XRD pattern of an AB-staggered mode offset by a distance of *a*/2 and *b*/2 (Fig. 2a, orange curve) does not reproduce the experimentally observed profile. The AB-staggered mode completely covers the pores (Fig. 2d,e). Because of the AA stacking structure, the H_2_TPP and NiPc units are alternately connected at identical intervals of 1.8 nm along both the *a* and *b* directions. The porphyrin and phthalocyanine sequence in the framework is •••H_2_TPP–NiPc–H_2_TPP–NiPc ••• for one grid line and is reversed to •••NiPc–H_2_TPP–NiPc–H_2_TPP ••• for the adjacent grid line (Fig. 1c). These primary structural periodicities in the *ab* plane extend to the stacking *c* dimension and enable control over high-order structures with periodic porphyrin-over-porphyrin and phthalocyanine-over-phthalocyanine columnar arrays and 1D microporous channels (Fig. 1c).

To gain insight into the structures, we applied the DFTB method to calculate the optimised geometries and orbital energies of the 2D materials (Supplementary Table 1). In the monolayer structure, the porphyrin and phthalocyanine rings are in the same plane, and the phenyl units are twisted with a dihedral angle of 64° relative to the porphyrin plane (Supplementary Fig. 5). In the stacked structures, we calculated three typical cases in which the same isomers aggregate on top of each other: the eclipsed AA, AA-slipped, and AB-staggered stacking modes. Simulations using an AA-slipped structure beginning at a slipping distance of *a*/32 between the layers eventually converged to the eclipsed AA stacking structure. Eclipsed AA stacking is rarely observed for COFs. The strong π-π interactions between the layers drive the formation of this highly ordered unique structure. In the frameworks, the dihedral angle between the phenyl unit and the porphyrin plane has significantly decreased to 41° (Supplementary Fig. 4), which indicates that the interlayer interactions overcome the steric repulsion between the *ortho*-hydrogen atoms. The eclipsed AA stacking mode has a total crystal stacking energy of 167.02 kcal mol^−1^ per unit cell per layer (Supplementary Table 1), which is 60 kcal mol^−1^ more stable than the AB-staggered structure (124.59 kcal mol^−1^).

### 2D antennae diversity

To demonstrate the scope of the *C*_4_ + *C*_4_ diagram, we explored the combination of freebase porphyrin with copper phthalocyanine (CuPc[OH]_8_, a IB metal) to replace the VIII metal derivative (i.e., NiPc[OH]_8_). Then, the freebase porphyrin was replaced with a zinc porphyrin (ZnTP_BA_P, a IIB metal), which was used in the polycondensation reaction with CuPc[OH]_8_ and NiPc[OH]_8_. Finally, copper porphyrin (CuTP_BA_P, a IB metal) was investigated for polycondensation with CuPc[OH]_8_ and NiPc[OH]_8_. Therefore, a significant feature of the *C*_4_ + *C*_4_ diagram is that this strategy is compatible with various metalloporphyrins and metallophthalocyanines for the construction of highly ordered microporous 2D materials (Fig. 1b). We demonstrated this concept by synthesising five different COFs; H_2_TPP-CuPc-COF, ZnTPP-NiPc-COF, ZnTPP-CuPc-COF, CuTPP-NiPc-COF, and CuTPP-CuPc-COF were synthesised in 75–82% isolated yields (see methods in Supplementary Information). IR spectroscopy results confirmed the formation of boronate ester linkages between two π-macrocycles (Supplementary Fig. 1). FE-SEM images revealed a flake-like or disk-like morphology with micrometer sizes (Supplementary Fig. 2). HR-TEM measurements indicated a tetragonal texture with parallel-aligned grid lines that were spaced at 1.8-nm intervals (Supplementary Fig. 3).

Although their central metals are different, the XRD peak positions and relative intensities of these microporous porphyrin-*co*-phthalocyanine 2D materials assigned to the (110), (200), (400), and (001) facets were similar to each other (Fig. 2a). These results indicate that, irrespective of the metal species, porphyrin and phthalocyanine are linked in the same AA-stacking lattice structure. Therefore, the new *C*_4_ + *C*_4_ topological design enables the construction of highly ordered porphyrin-*co*-phthalocyanine 2D materials, and the two monomers can be predesigned with different central metal species, leading to broad 2D material diversity.

### Gas adsorption

**Figure 3:**
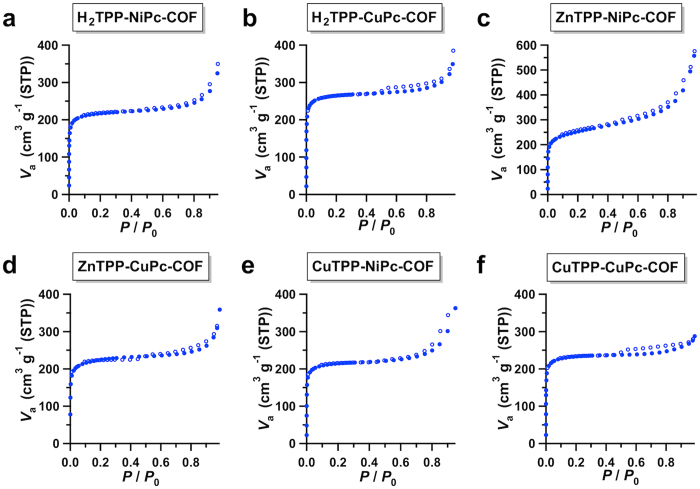
Gas adsorption of microporous 2D porphyrin-*co*-phthalocyanine COFs. (**a**–**f**) Typical nitrogen sorption isotherm curves of the microporous M_1_TPP-M_2_Pc-COFs measured at 77 K (filled circles for adsorption and open circles for desorption).

To investigate the porosity, we conducted nitrogen sorption isotherm measurements at 77 K. These COFs exhibited reversible profiles with typical type I characteristics (Fig. 3a–f), indicating that they are microporous materials. Notably, these 2D materials possess high Brunauer–Emmett–Teller (BET) surface areas that range from 693 to 940 m^2^ g^−1^ (Table 1). Their pore volumes were determined to be 0.5–0.6 cm^3 ^g^−1^ (Supplementary Fig. 5). The pore size and size distribution profiles revealed the existence of only one type of micropore that was 1.8 nm in size, which is consistent with the theoretical pore size (Supplementary Fig. 5).

### *C*_4_ + *C*_2_ diagram and mesoporous porphyrin-*co*-phthalocyanine 2D antennae

The highly ordered porphyrin-*co*-phthalocyanine 2D antennae are not limited to microporous structures. We further extended our strategy to the exploration of highly ordered mesoporous porphyrin-*co*-phthalocyanine 2D antennae. For this purpose, we employed a *C*_4_ + *C*_2_ geometry diagram. The *C*_4_ tetraphenyl porphyrin (i.e., M_1_TP_BA_P) was replaced with a *C*_2_-symmetric diphenyl porphyrin (Fig. 1d, M_1_DP_BA_P) for the polycondensation reaction with M_2_Pc[OH]_8_. As shown in Fig. 1d, in the *C*_4_ + *C*_2_ topological diagram, the phthalocyanine units remain at the vertices, whereas the porphyrin moieties move to the edge positions that connect the phthalocyanine vertices. In this structure, the grid line separation and pore size were expanded to 3.6 nm, which is twice that of the microporous 2D antennae (Fig. 1). The mesoporous porphyrin-*co*-phthalocyanine 2D antennae have a 2:1 porphyrin-to-phthalocyanine stoichiometric ratio and consist of a sequence of π-macrocycles in the order M_1_ – M_2_ – M_1_ – M_2_ – ••• for all of the grid lines from both the *a* and *b* directions (Fig. 1d and Table 1).

The *C*_4_ + *C*_2_ topological diagram is also compatible with a variety of metalloporphyins and metallophthalocyanines with different central metals. We demonstrated this feature by developing the polycondensation reaction of M_1_DP_BA_P and M_2_Pc[OH]_8_ in a 2/1 (mole/mole) stoichiometric ratio under solvothermal conditions in DMAc/*o*-DCB (2/1 v/v), and we isolated M_1_DPP-M_2_Pc-COFs as black solids in 75–85% yields (Fig. 1, M_1_DPP-M_2_Pc-COFs, M_1_ = H_2_, Zn, Cu; M_2_ = Ni, Cu). A different COF that containing a cobalt phthalocyanine unit was prepared. However, this COF exhibited rather low crystallinity and its optical and catalytic functions were unclear^28^. The IR spectra confirmed the formation of the boronate ester linkages (Supplementary Fig. 6). The mesoporous materials exhibited a belt-shaped morphology with sizes on the micrometer scale, as revealed by FE-SEM (Supplementary Fig. 7). Their tetragonal porous textures with regularly ordered grid lines at a discrete separation of 3.6 nm were observed in the HR-TEM images (Supplementary Fig. 8); these results confirm the formation of a highly ordered lattice structure.

### Mesoporous lattice structure

**Figure 4:**
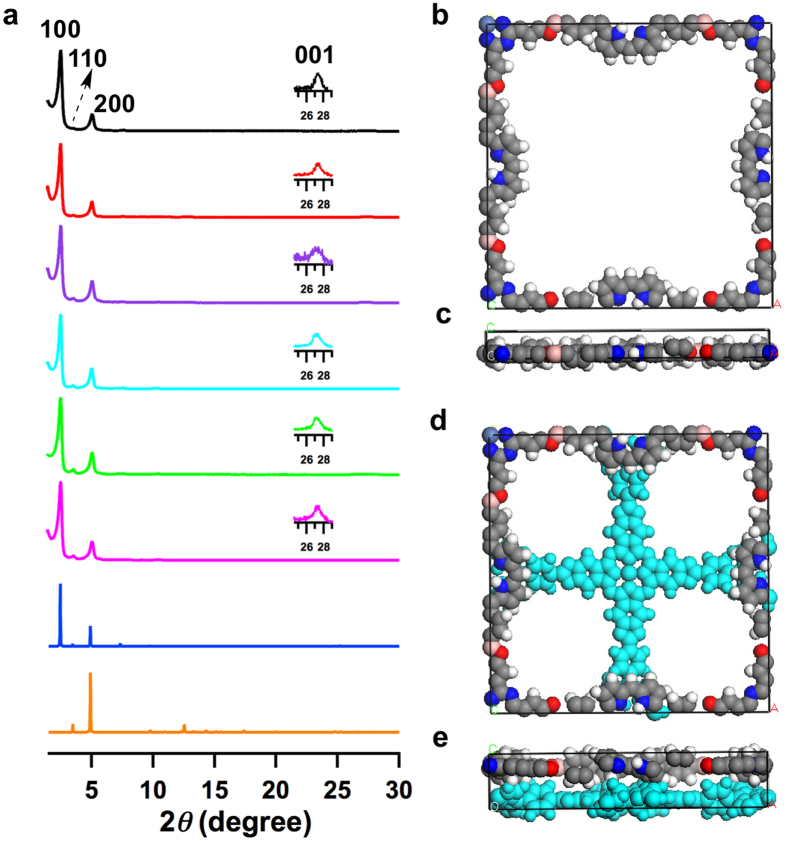
Crystalline structures of mesoporous 2D porphyrin-*co*-phthalocyanine COFs. (**a**) Experimentally observed XRD patterns (black curve for H_2_DPP-NiPc-COF, red curve for H_2_DPP-CuPc-COF, purple curve for ZnDPP-NiPc-COF, sky-blue curve for ZnDPP-CuPc-COF, green curve for CuDPP-NiPc-COF and magenta curve for CuDPP-CuPc-COF) and patterns simulated with the eclipsed AA stacking (blue curve) for H_2_DPP-NiPc-COF and with the AB-staggered stacking (orange curve) for H_2_DPP-NiPc-COF. (**b**,**c**) Views of the unit cell along the *z* (**b**) and *y* (**c**) axes of H_2_DPP-NiPc-COF. (**d**,**e**) Views of the AB-staggered unit cell along the *z* (**d**) and *y* (**e**) axes. Insets in XRD profiles show the enlarged views of the (001) peaks.

All of the M_1_DPP-M_2_Pc-COFs exhibited very strong XRD peaks with similar diffraction positions and intensities (Fig. 4a). Consistently with the DFTB-reconstructed structure, the peak at 2*θ* = 2.5° is assigned to the (100) facet with a grid line separation of 3.6 nm. The peaks at 3.5° and 5.0° correspond to the (110) and (200) facets, respectively. The presence of the (001) facet, as evident by the peak at 2*θ* angle of 27°, indicates that the π-units in the M_1_DPP-M_2_Pc-COFs are periodic in all three dimensions. The interlayer distance was determined to be 3.4 Å. As exemplified by H_2_DPP-NiPc-COF, the eclipsed AA-stacking structure produced a simulated XRD pattern (Fig. 4a, blue curve), in which the peak positions and intensitiesis are consistent with the experimental values (Fig. 4a, black curve). The 2D sheets stack to form periodically ordered and unidirectionally stacked porphyrin-over-porphyrin and phthalocyanine-over-phthalocyanine columnar arrays (Figs 1e and 4b,c). In sharp contrast, the AB-staggered arrangement does not match the experimentally observed patterns (Fig. 4a, orange curve) and results in the pore overlap (Fig. 4d,e).

In the case of H_2_DPP-NiPc-COF, for example, the diphenyl porphyrin unit was slightly distorted to adopt a saddle conformation in the monolayer with a dihedral angle between the phenyl group and the plane of the porphyrin units of 45°. In the layered structure, this dihedral angle decreased to 22° (Supplementary Fig. 9). The eclipsed AA stacking mode has a total crystal stacking energy of 133.71 kcal mol^−1^ per unit cell per layer (Supplementary Table 1), which is 53 kcal mol^−1^ greater than that of the AB-staggered structure (80.36 kcal mol^−1^). Notably, because of its dense π-units in the lattice, the total crystal stacking energy of microporous H_2_TPP-NiPc-COF (167.02 kcal mol^−1^) is much higher than that of mesoporous H_2_DPP-NiPc-COF (133.71 kcal mol^−1^).

### Gas adsorption

**Figure 5:**
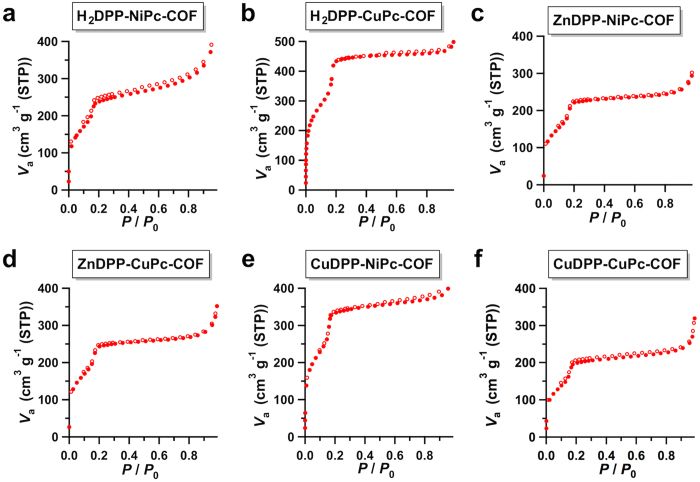
Gas adsorption of mesoporous 2D porphyrin-*co*-phthalocyanine COFs. (**a**–**f**) Typical nitrogen sorption isotherm curves of the mesoporous M_1_DPP-M_2_Pc-COFs measured at 77 K (filled circles for adsorption and open circles for desorption).

The nitrogen sorption isotherm curves for M_1_DPP-M_2_Pc-COFs exhibit typical type IV behaviour (Fig. 5), which is characteristic of mesoporous materials. The BET surface areas were evaluated to be as high as 660, 705, 719, 840, 1031, and 1282 m^2^ g^−1^ for CuDPP-CuPc-COF, ZnDPP-NiPc-COF, ZnDPP-CuPc-COF, H_2_DPP-NiPc-COF, CuDPP-NiPc-COF, and H_2_DPP-CuPc-COF, respectively (Table 1). The high BET surface area of H_2_DPP-CuPC-COF is related to its high crystallinity. The pore volumes ranged between 0.4 and 0.9 cm^3^ g^−1^ (Supplementary Fig. 10). Evaluation of the sorption curves revealed the presence of only one type of mesopore, with a pore width of 3.6 nm (Supplementary Fig. 10).

### Light-harvesting functions

**Figure 6:**
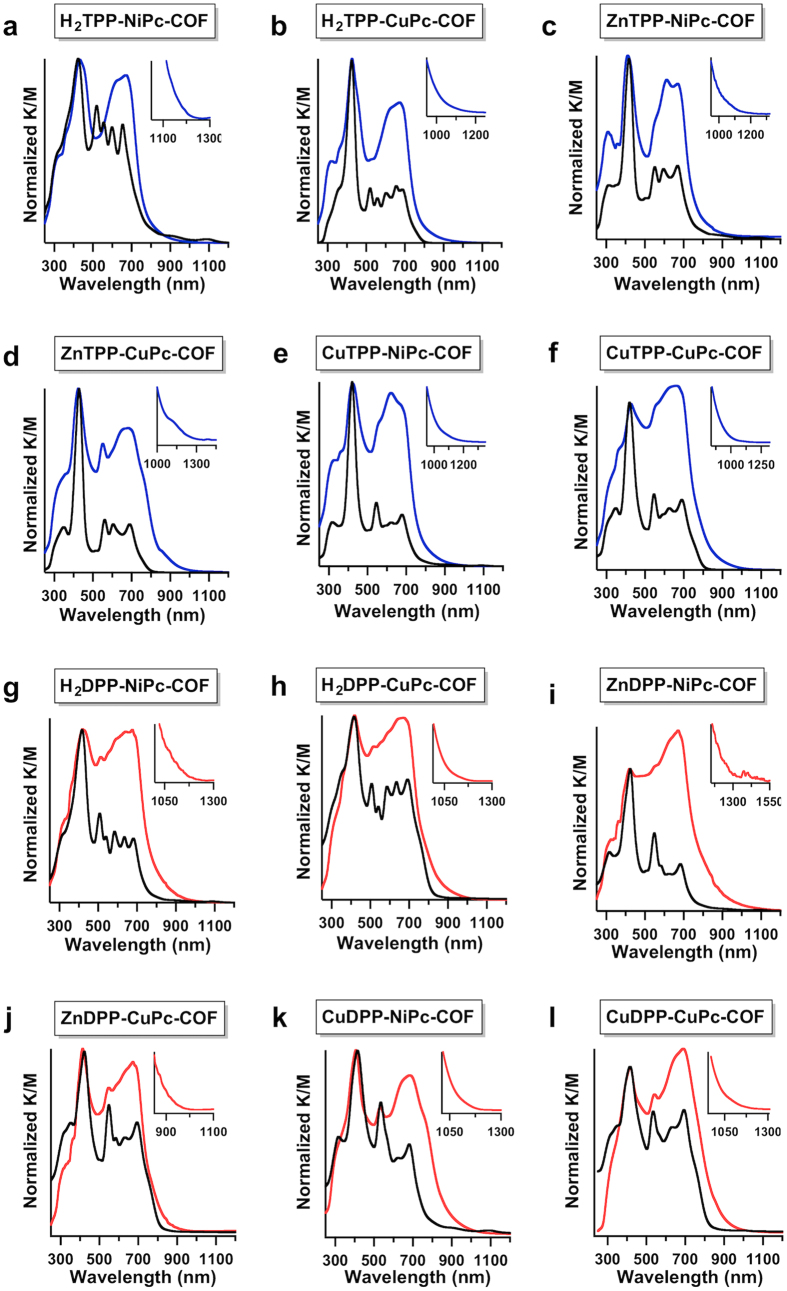
Light-harvesting functions. (**a**–**f**) Absorption spectra of the microporous M_1_TPP-M_2_Pc-COFs (blue curves) and their corresponding monomer-mixture controls (black curves). (**g**–**l**) Absorption spectra of the mesoporous M_1_DPP-M_2_Pc-COFs (red curves) and their corresponding monomer-mixture controls (black curves). Insets show the onset absorption spectra.

The highly ordered porphyrin-*co*-phthalocyanine 2D antennae exhibited a synergistic effect for merging the light-absorption function of porphyrin and phthalocyanine to achieve remarkable optical and electronic properties. To evaluate the light-absorption functions of the porphyrin-*co*-phthalocyanine 2D antennae, we collected their electronic absorption spectra (Fig. 6). In contrast to the single-component porphyrin COFs and phthalocyanine COFs that exhibit major absorbance in either the visible or the near-infrared region^19^,^22^, the highly-ordered porphyrin-*co*-phthalocyanine 2D antennae exhibit a strong absorption capacity in both the visible and near-infrared regions. For a comparative study, mixtures consisting of porphyrin (M_1_DP_BE_P or M_1_TP_BE_P; BE = boronate ester) and phthalocyanine (M_2_Pc[MeO]_8_) monomers were used as a control; these mixtures contained the same components as the 2D COFs (M_1_DP_BE_P/M_2_Pc[MeO]_8_ = 2/1 mole/mole for M_1_DPP-M_2_Pc-COFs and M_1_TP_BE_P/M_2_Pc[MeO]_8_ = 1/1 mole/mole for M_1_TPP-M_2_Pc-COFs). The absorption spectra nomarlised at the Soret band of porphyrins are shown in Fig. 6. In comparison to the monomer mixture controls, the highly ordered microporous porphyrin-*co*-phthalocyanine 2D COFs exhibited exceptionally high light-absorption capacities over a wide solar spectrum from 300 to longer than 900 nm (Fig. 6a–f). Another remarkable feature is that these 2D antennae are exceptionally strong absorbers of near-infrared light. For example, the relative intensity ratio (*I*_Q_/*I*_S_)_COFs_ of the Q band (685 nm) to the Soret band (420 nm) was 0.90 for mesoporous H_2_TPP-NiPc-COF, whereas the (*I*_Q_/*I*_B_)_control_ value of its control was 0.64, indicating a 1.4-fold enhancement according to the ratio (*I*_Q_/*I*_S_)_COFs_/(*I*_Q_/*I*_S_)_control_ (Table 1). Notably, all of the other microporous M_1_TPP-M_2_Pc-COFs exhibited a similar trend. The (*I*_Q_/*I*_S_)_COFs_/(*I*_Q_/*I*_S_)_control_ values ranged from 2.1 to 3.3 (Table 1). These enhanced absorption capacities are related to the highly ordered stacking structures, which is consistent with Kasha’s prediction regarding the effect of H-aggregates on the light absorption of π-macrocycles^22^. The Q-band absorption enhancement has been observed for single-component phthalocyanine COFs^22^.

The mesoporous porphyrin-*co*-phthalocyanine 2D antennae also exhibited an enhanced light-harvesting capacity over a broad solar spectrum (Fig. 6g–l). In these COFs, the (*I*_Q_/*I*_S_)_COFs_/(*I*_Q_/*I*_S_)_control_ values were 1.5–3.8 (Table 1). Therefore, the highly ordered porphyrin-*co*-phthalocyanine antennae exhibited dramatically enhanced light-harvesting capacities, which were four fold larger than those of the unstructured materials.

The ordered 2D materials extended the absorbance to long-wavelength regions (Fig. 6 and insets, Table 1). All of the microporous antennae exhibited the same trend (Fig. 6a–f and insets, Table 1), and ZnTPP-CuPc-COF exhibited the longest onset absorbance of 1350 nm. Among the mesoporous antennae, the ZnDPP-NiPc-COF exhibited the longest onset absorption at 1322 nm. On the basis of these absorption onsets, we evaluated their optical band gaps (Table 1). For example, in the microporous series, the smallest and largest band gaps were 0.92 eV and 1.13 eV, respectively. Among the mesoporous antennae, the smallest band gap was only 0.93 eV, and the largest one was 1.23 eV. The band gap of organic π-systems determines their functions. A low band gap facilitates electron motion from the valence band to the conduction band and enables the utilisation of low-energy photons to generate photoexcited states. Therefore, these ordered light-harvesting 2D antennae with broad absorption spectra and low band gaps open a new era of solar energy utilisation. In this study, we explored their photosensitising activity for the activation of triplet molecular oxygen.

## Discussion

Singlet oxygen (^1^O_2_; ^1^Δ_g_) is highly desired for photodynamic therapy because of its high oxidative capability^36^,^37^,^38^,^39^,^40^,^41^. A photosensitiser is required to generate singlet oxygen because the direct excitation from a triplet molecular oxygen (^3^O_2_; ^3^Δ_g_) to a singlet ^1^O_2_ is forbidden^42^,^43^,^44^,^45^,^46^,^47^. Upon light absorption, the photosensitiser generates an excited triplet state (T_1_) via intersystem crossing from excited singlet state (S_1_); the triple excited state further transfers the excitation energy to ^3^O_2_ and produces ^1^O_2_. A variety of photosensitisers have been investigated for the generation of singlet oxygen, including rose bengal, fluorescein, porphyrin, and phthalocyanine^42^,^46^,^47^. Because of their high singlet-oxygen quantum yield and strong visible-light absorption ability, the most widely used photosensitisers are porphyrin derivatives and some of these derivatives have been commercialised for photodynamic therapy (i.e., Foscan^48^, Laser-phyrin^49^, Photofrin^50^, and Visudyne^51^). However, the production of ideal photosensitisers for photodynamic therapy with a much greater efficiency and light-harvesting capacity in the long-wavelength region remains challenging. We investigated singlet oxygen production using these highly ordered porphyrin-*co*-phthalocyanine 2D antennae as a photosensitiser (Supplementary Information).

**Figure 7:**
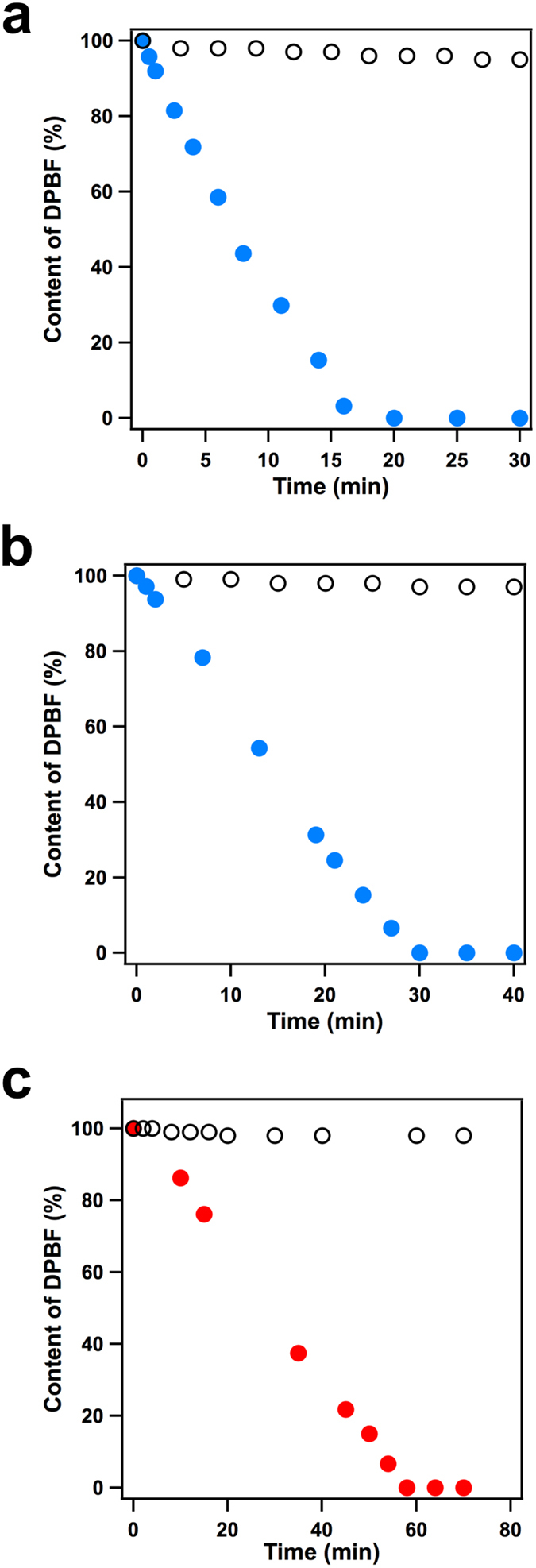
Singlet oxygen generation. (**a**) Plot of the DPBF content in the presence of ZnTPP-CuPc-COF (blue dots) and in the presence of a mixture of ZnTPP and CuPc (black circles) as a function of time, under irradiation of 500-nm light. (**b**) Plot of the DPBF content in the presence of ZnDPP-CuPc-COF (blue dots) and in the presence of a mixture of ZnDPP and CuPc (black circles) as a function of time, under irradiation of 500-nm light. (**c**) Plot of the DPBF content in the presence of ZnDPP-CuPc-COF (red dots) and in the presence of a mixture of ZnDPP and CuPc (black circles) as a function of time, under irradiation of 750-nm light.

The generation of singlet oxygen was investigated using the well-established singlet oxygen label 1,3-diphenylisobenzofuran (DPBF) upon irradiation at 500 nm; this process was monitored by time-dependent electronic absorption spectroscopy^52^. The light irradiation of an oxygen-saturated *o*-DCB solution (2.3 mL) containing DPBF (50 *μ*M) in the presence of the ZnTPP-CuPc-COF (0.5 mg) resulted in the steady generation of singlet oxygen, which was confirmed by a characteristic spectral change (Supplementary Fig. 11a). In contrast, a control sample consisting of a mixture of ZnTPP and CuPc with the same composition as the COF exhibited a very low activity in the same reaction, resulting in only a small spectral change (Supplementary Fig. 11b). The time-dependent plot indicated that the content of DPBF in the presence of the ZnTPP-CuPc-COF decreased rapidly (Fig. 7a, blue dots). However, the system with the monomer mixture is very slow (Fig. 7a, black circles). Remarkably, the catalytic activity of the ZnTPP-CuPc-COF is more than three orders of magnitude greater than that of the mixture control. Compared to the single-component porphyrin COFs, the porphyrin-*co*-phthalocyanine COFs show one order of magnitude higher activity^24^. The generation of singlet oxygen requires the excited triplet state of a photosensitiser. Therefore, upon irradiation, the ZnTPP-CuPc-COF can efficiently generate the excited triplet state, which triggers the activation of triplet molecular oxygen to produce singlet oxygen. The difference between the control and COF suggests that the ordered π-structure is key to the light-induced generation of triplet-excited states.

The photocatalytic functions of the mesoporous ZnDPP-CuPc-COF were also investigated. The ZnDPP-CuPc-COF induced the typical spectral change of DPBF upon light irradiation (Supplementary Fig. 11c). The ZnDPP-CuPc-COF exhibited nearly 1000-fold enhanced photocatalytic activity compared to that of the monomer mixture (Fig. 7b, Supplementary Fig. 11d). Irradiation of the porphyrin-*co*-phthalocyanine 2D materials with 500-nm light resulted in excitation of the zinc porphyrin columns. Metalloporphyrins with noble-metal species, such as palladium and platinum, have been extensively explored for the generation of singlet oxygen because of their high triplet-state efficiency. Therefore, the highly ordered porphyrin-*co*-phthalocyanine 2D antennae, which do not contain noble metals, represent a new type of photosensitisers.

The investigation of long-wavelength light for the production of singlet oxygen is important for photodynamic phototherapy because red light can reach deeper regions. We further explored the utilisation of 750-nm light for the production of singlet oxygen. Upon irradiation with 750-nm light, the ZnDPP-CuPc-COF catalysed the generation of singlet oxygen, as shown by the spectral change of DPBF (Supplementary Fig. 11e). The time-dependent plot indicates that the catalytic reaction proceeds steadily and is completed within 60 min (Fig. 7c, red dots). In contrast, the control monomer mixture exhibited rather low activity for the photogeneration of singlet oxygen (Fig. 7c, black circles, Supplementary Fig. 11f). Upon irradiation, single-component phthalocyanine COFs have been reported to produce a triplet excited state via intersystem crossing from a photoexcited singlet state^33^. The 750-nm irradiation excites the phthalocyanine columns in the 2D antennae, which generate a triplet-excited state that transfers its excitation energy to triplet molecular oxygen. To use long-wavelength near infrared light, the state-of-the-art technique involves two-photon absorption systems driven by tense pulse laser^53^. Therefore, the highly ordered porphyrin-*co*-phthalocyanine 2D light-harvesting antennae are unique in that they can directly utilise infrared light for the production of singlet oxygen, which provides new access to this fundamental yet challenging photoreaction. These 2D π-electronic COFs are multiple functional materials and are interesting platform for construction of photoinduced electron transfer systems. The adsorption over 800 nm of these COFs is useful for harvesting near IR light for photoenergy conversions such as in solar cells, which is worthy of further investigation.

## Conclusion

We have developed a general strategy for designing highly ordered porphyrin-*co*-phthalocyanine 2D light-harvesting antennae via topology-directed polycondensation. This strategy exploits covalent bonds for the construction of 2D polymer sheets and noncovalent forces for crystallisation of 2D polymers into layered structures, which lead to the creation of periodic porphyrin and phthalocyanine columns and ordered 1D nanochannels. The newly developed *C*_4_ + *C*_4_ diagram, in conjunction with the *C*_4_ + *C*_2_ diagram, enables the predesign of not only the primary structure of 2D polymers but also the high-order structure of the columnar array and channel size. These topological diagrams are compatible with various metalloporphyrins and metallophthalocyanines, which allows for a high flexibility in designing different metal species. The porphyrin-*co*-phthalocyanine 2D antennae not only possess discrete micropores or mesopores but also exhibit unique light-harvesting functions and photocatalytic properties that are not available with non-ordered or single-component COF materials. The ordered 2D light-harvesting antennae with superb absorbance and low band gaps open a new era of solar energy utilisation ranging from photocatalysis to photoenergy conversion.

## Methods

### Synthesis of H_2_TPP-NiPc-COF

A mixture of H_2_TP_BA_P (23.7 mg, 0.03 mmol) and NiPc[OH]_8_ (21.0 mg, 0.03 mmol) in DMAC/*o*-DCB (2 mL, 1/1 v/v) was degassed in a Pyrex tube (10 mL) by using three freeze–pump–thaw cycles. The tube was sealed and heated at 120 °C for 7 days. The precipitate was collected by centrifugation, washed with anhydrous DMAc and anhydrous acetone 5 times, extracted using a Soxhlet extractor with anhydrous acetone for 3 days, and dried at 150 °C under vacuum for 24 h to yield a black solid in 76% isolated yield. Other microporous M_1_TPP-M_2_Pc-COFs were synthesised under similar conditions, as shown in Supplementary synthetic procedures.

### Synthesis of H_2_DPP-NiPc-COF

A mixture of H_2_DP_BA_P (33.1 mg, 0.06 mmol) and NiPc[OH]_8_ (21.0 mg, 0.03 mmol) in DMAC/*o*-DCB (2 mL, 2/1 v/v) was degassed in a Pyrex tube (10 mL) by using three freeze–pump–thaw cycles. The tube was sealed and heated at 120 °C for 7 days. The precipitate was collected by centrifugation, washed with anhydrous DMAc and anhydrous acetone 5 times, extracted using a Soxhlet extractor with anhydrous acetone for 3 days, and dried at 150 °C under vacuum for 24 h to yield a black solid in 75% isolated yield. Other mesoporous M_1_DPP-M_2_Pc-COFs were synthesised under similar conditions, as shown in the Supplementary synthesis procedures.

## Additional Information

**How to cite this article**: Feng, X. *et al*. Two-dimensional artificial light-harvesting antennae with predesigned high-order structure and robust photosensitising activity. *Sci. Rep.* **6**, 32944; doi: 10.1038/srep32944 (2016).

## Supplementary Material

Supplementary Information

## Figures and Tables

**Table 1:** Properties of 2D porphyrin-*co*-phthalocyanine COFs.

2D COFs	Stoichiometry (TPP/Pc or DPP/Pc)	Pore Size & Grid Line Separation (nm)	BET Surface Area (m^2^ g^−1^)	Sequence in Grid Lines	(*I*_Q_/*I*_S_)_COFs_/(*I*_Q_/*I*_S_)_control_	Absorption Onset (nm)	Band Gap (eV)
H_2_TPP-NiPc	1/1	1.8	702	H_2_–Ni••• Ni–H_2_•••	1.4	1252 (986)^a^	0.99 (1.26)^b^
H_2_TPP-CuPc	1/1	1.8	940	H_2_–Cu••• Cu–H_2_•••	2.7	1097 (836)	1.13 (1.48)
ZnTPP-NiPc	1/1	1.8	815	Zn–Ni••• Ni–Zn•••	2.1	1214 (1024)	1.02 (1.21)
ZnTPP-CuPc	1/1	1.8	701	Zn–Cu••• Cu–Zn•••	3.0	1350 (894)	0.92 (1.38)
CuTPP-NiPc	1/1	1.8	693	Cu–Ni••• Ni–Cu•••	3.3	1200 (1021)	1.03 (1.21)
CuTPP-CuPc	1/1	1.8	714	Cu–CuPc••• CuPc–Cu•••	3.3	1177 (1038)	1.05 (1.20)
H_2_DPP-NiPc	2/1	3.6	840	H_2_–Ni•••	2.7	1189 (1020)	1.04 (1.21)
H_2_DPP-CuPc	2/1	3.6	1282	H_2_–Cu•••	1.5	1158 (1082)	1.07 (1.15)
ZnDPP-NiPc	2/1	3.6	705	Zn–Ni•••	3.8	1322 (1223)	0.93 (1.01)
ZnDPP-CuPc	2/1	3.6	719	Zn–Cu•••	1.5	1004	1.23 (1.27)
CuDPP-NiPc	2/1	3.6	1031	Cu–Ni•••	1.8	1193 (1017)	1.03 (1.22)
CuDPP-CuPc	2/1	3.6	660	Cu–Cu•••	1.5	1114 (988)	1.11 (1.26)

^a^The value in parentheses is the onset absorbance of the corresponding mixture controls. ^b^The value in parentheses is the gap of the corresponding mixture controls.
