# Supplementary material for: Two-dimensional artificial light-harvesting antennae with predesigned high-order structure and robust photosensitising activity
Source: Sci Rep. 2016 Sep 13;6:32944. doi: 10.1038/srep32944 (PMC5020651; doi:10.1038/srep32944)
Supplement: Supplementary Information [file srep32944-s1.pdf]

Supporting Information

Two-dimensional artificial light-harvesting antennae with predesigned high-order structure and robust photosensitising activity

Xiao Feng[1,2], Xuesong Ding[1], Long Chen[1,2], Yang Wu[1], Lili Liu[3], Matthew Addicoat[3], Stephan Irle[3], Yuping Dong[2] & Donglin Jiang[1]

[1]*Field of Environment and Energy, School of Materials Science, Japan Advanced Institute of Science and Technology, 1-1 Asahidai, Nomi 923-1292, Japan.*

[2]*College of Materials Science and Engineering, Beijing Institute of Technology, Zhongguancun South Street, Beijing, 100081, China.*

[3]*WPI-Research Initiative-Institute of Transformative Bio-Molecules and Department of Chemistry, Graduate School of Science, Nagoya University, Furo-cho, Chikusa-ku, Nagoya 464-8602, Japan.*

E-mail: djiang@jaist.ac.jp

Supplementary Table 1. Lattice parameters of COFs

COFs	$a = b$ (Å)	c (Å)	Total DFTB Energy (ha)	LJ Energy (ha)	LJ Crystal Stacking Energy per unit cell (kcal/mol)	Total Crystal Stacking Energy per unit cell (kcal/mol)
**Monolayers**						
**H_2TPP-NiPc**	35.90		-410.719050	1.567069	–	–
**ZnTPP-NiPc**	35.88		-418.319113	1.558173	–	–
**H_2DPP-NiPc**	36.19		-254.296553	0.978355	–	–
**ZnDPP-NiPc**	35.89		-261.897847	0.966401	–	–
**AA stacking**						
**H_2TPP-NiPc-COF**	35.98	3.74	-821.970438	2.534110	188.26	167.02
**ZnTPP-NiPc-COF**	35.93	3.76	-837.182612	2.529015	184.28	170.80
**H_2DPP-NiPc-COF**	36.25	3.62	-509.019282	1.523212	136.01	133.71
**ZnDPP-NiPc-COF**	35.96	3.64	-524.210627	1.514792	131.15	130.19
**AA-slip stacking**						
**H_2TPP-NiPc-COF**	AA	–	–	–	–	–
**ZnTPP-NiPc-COF**	AA	–	–	–	–	–
**H_2DPP-NiPc-COF**	AA	–	–	–	–	–
**ZnDPP-NiPc-COF**	35.83	3.61	-524.216243	1.508896	133.00	131.95
**AB stacking**						
**H_2TPP-NiPc-COF**	36.00	3.76	-821.835197	2.752885	119.62	124.59
**ZnTPP-NiPc-COF**	35.66	3.32	-837.064329	2.684377	135.53	133.69
**H_2DPP-NiPc-COF**	36.25	3.48	-508.849236	1.704375	79.17	80.36
**ZnDPP-NiPc-COF**	36.00	3.53	-524.028138	1.702666	72.21	72.93

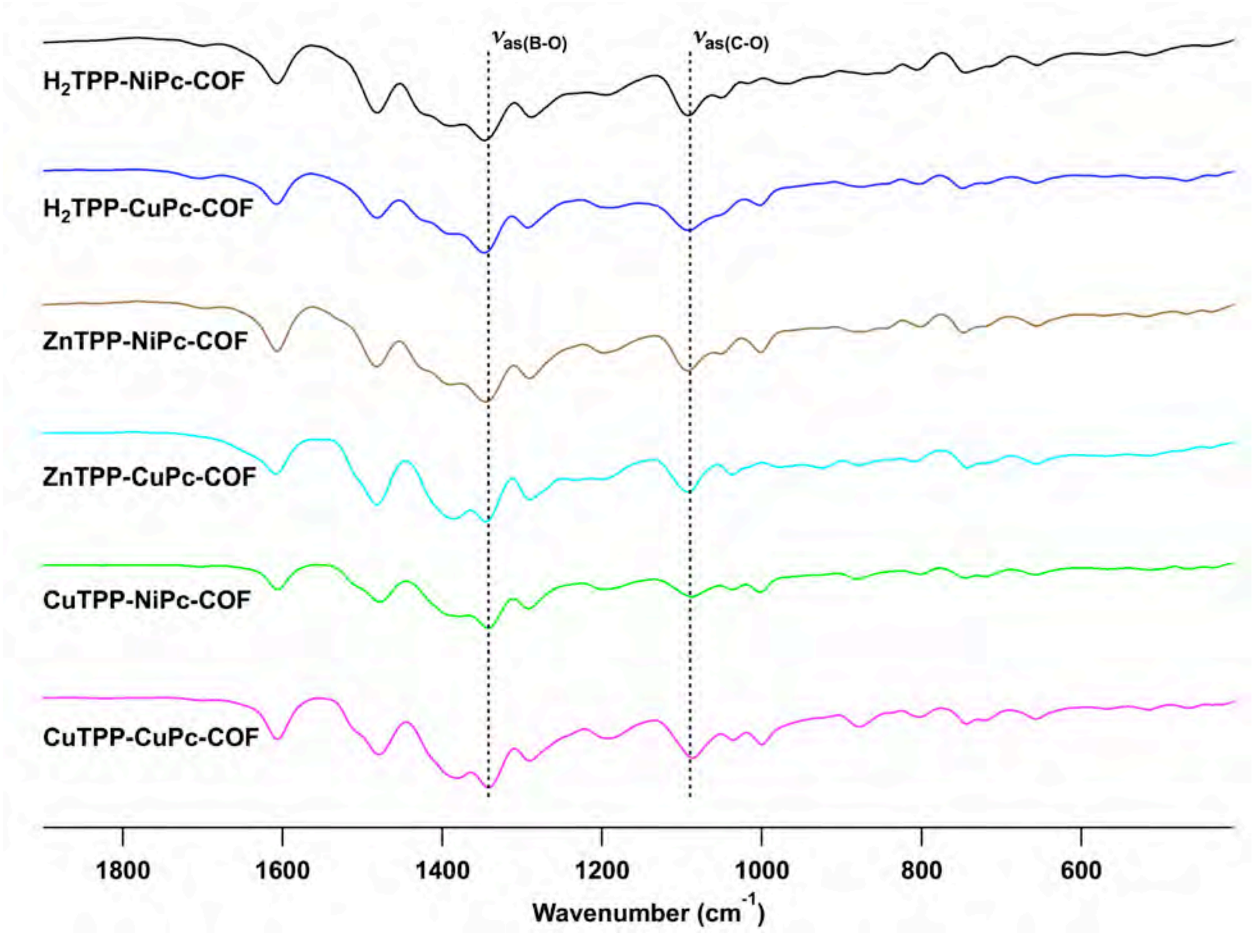

Supplementary Figure 1. IR spectra of H_2TPP-NiPc-COF, H_2TPP-CuPc-COF, ZnTPP-NiPc-COF, ZnTPP-CuPc-COF, CuTPP-NiPc-COF and CuTPP-CuPc-COF.

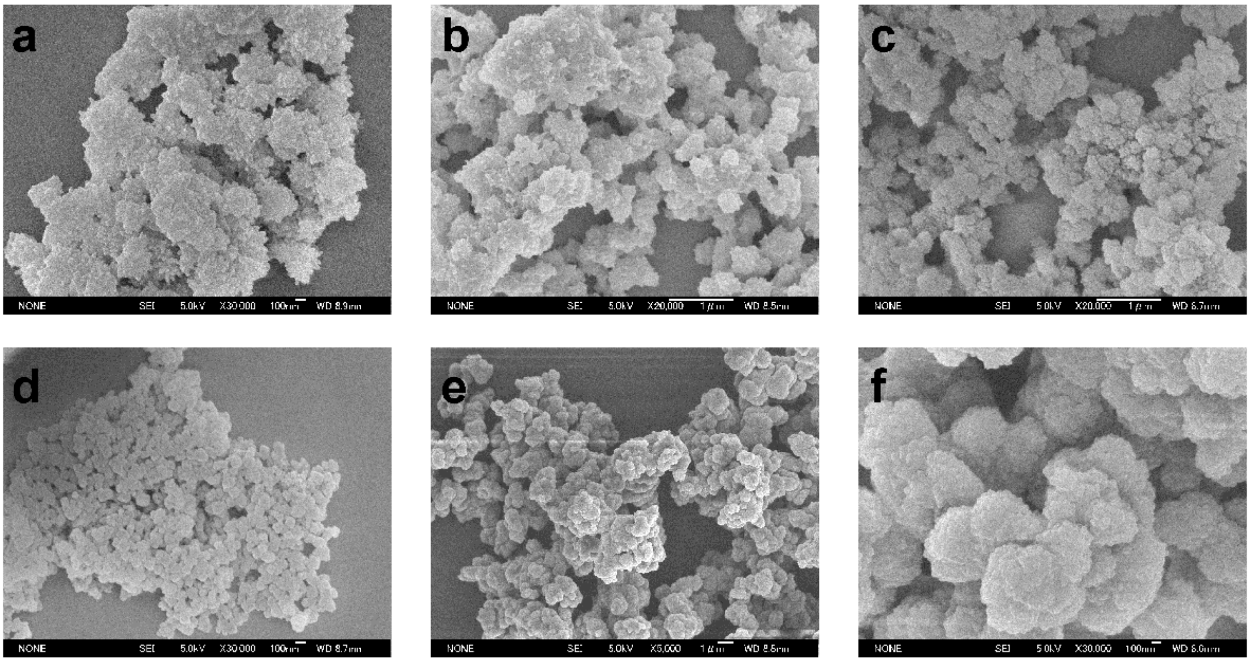

Supplementary Figure 2. FE-SEM images of (**a**) H_2TPP-NiPc-COF, (**b**) H_2TPP-CuPc-COF, (**c**) ZnTPP-NiPc-COF, (**d**) ZnTPP-CuPc-COF, (**e**) CuTPP-NiPc-COF and (**f**) CuTPP-CuPc-COF.

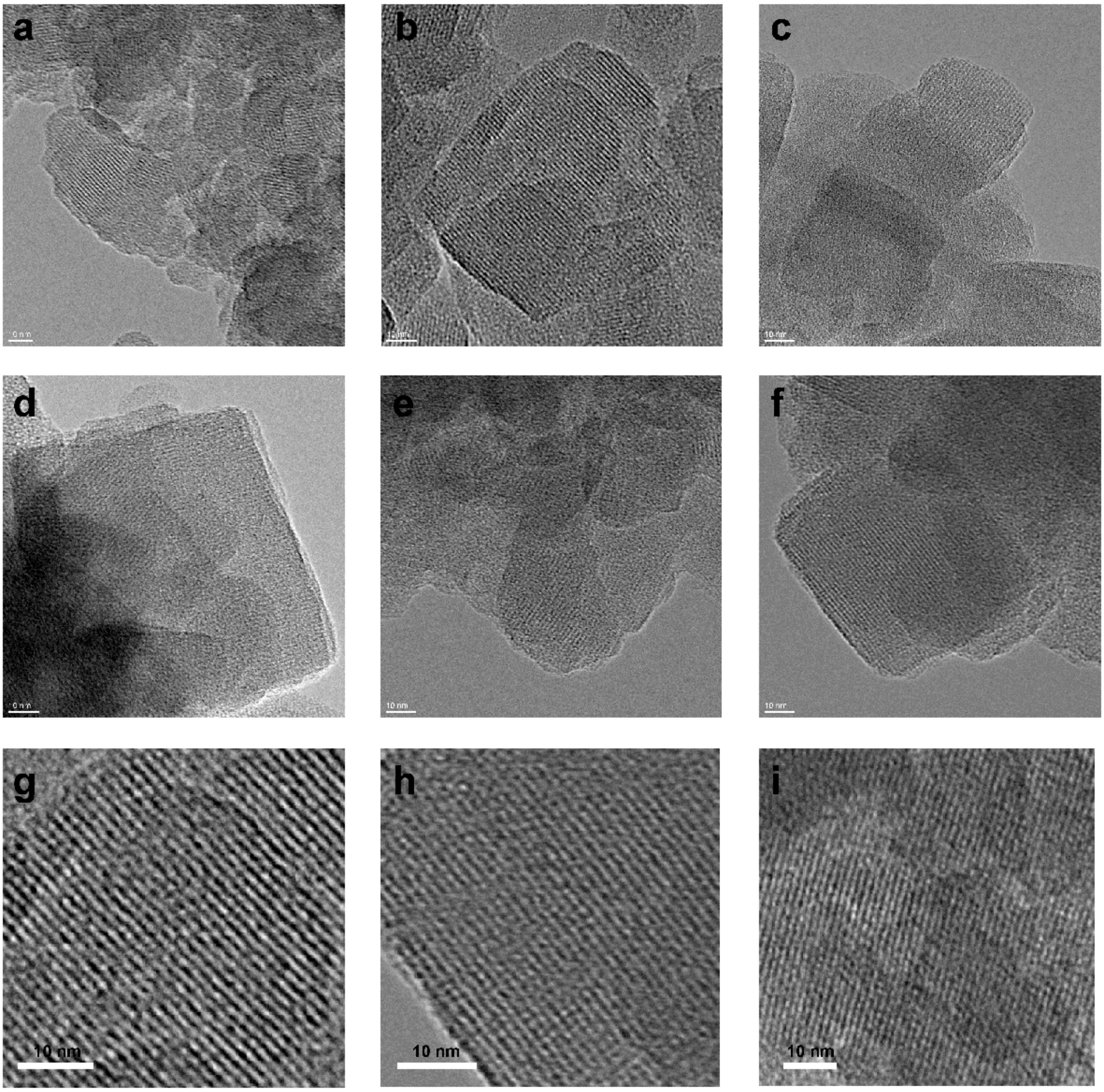

Supplementary Figure 3. HR-TEM images of (**a**) H_2TPP-NiPc-COF, (**b**) H_2TPP-CuPc-COF, (**c**) ZnTPP-NiPc-COF, (**d**) ZnTPP-CuPc-COF, (**e**) CuTPP-NiPc-COF and (**f**) CuTPP-CuPc-COF. (**g**)–(**i**) Enlarged HR-TEM images of (**g**) H_2TPP-NiPc-COF, (**h**) H_2TPP-CuPc-COF and (**i**) ZnTPP-NiPc-COF.

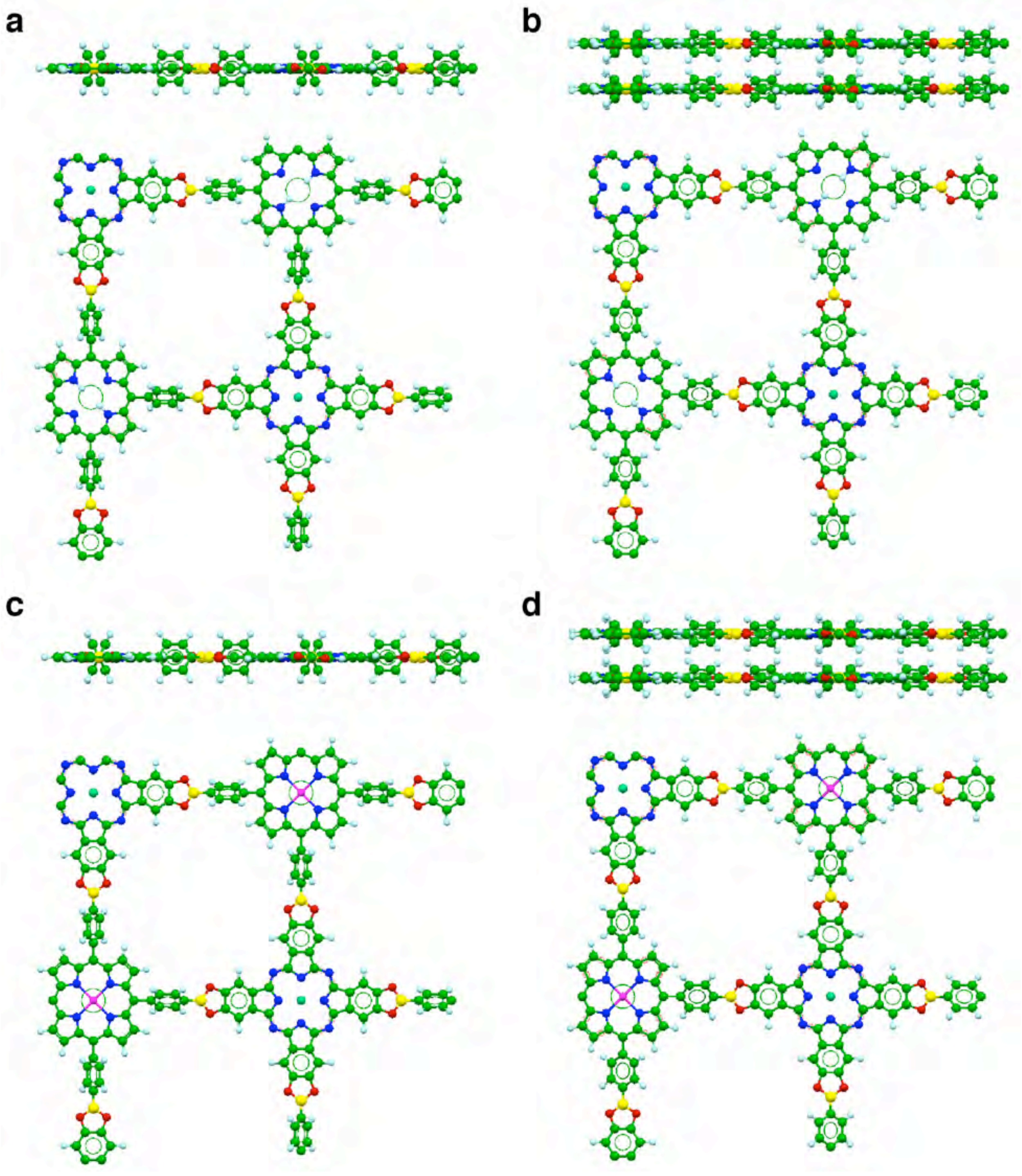

Supplementary Figure 4. (**a**) Optimized monolayer structure of H_2TPP-NiPc-COF along the *y* and *z* axes. (**b**) Optimized double-layer structure of H_2TPP-NiPc-COF along the *y* and *z* axes. (**c**) Optimized monolayer structure of ZnTPP-NiPc-COF along the *y* and *z* axes. (**d**) Optimized double-layer structure of ZnTPP-NiPc-COF along the *y* and *z* axes.

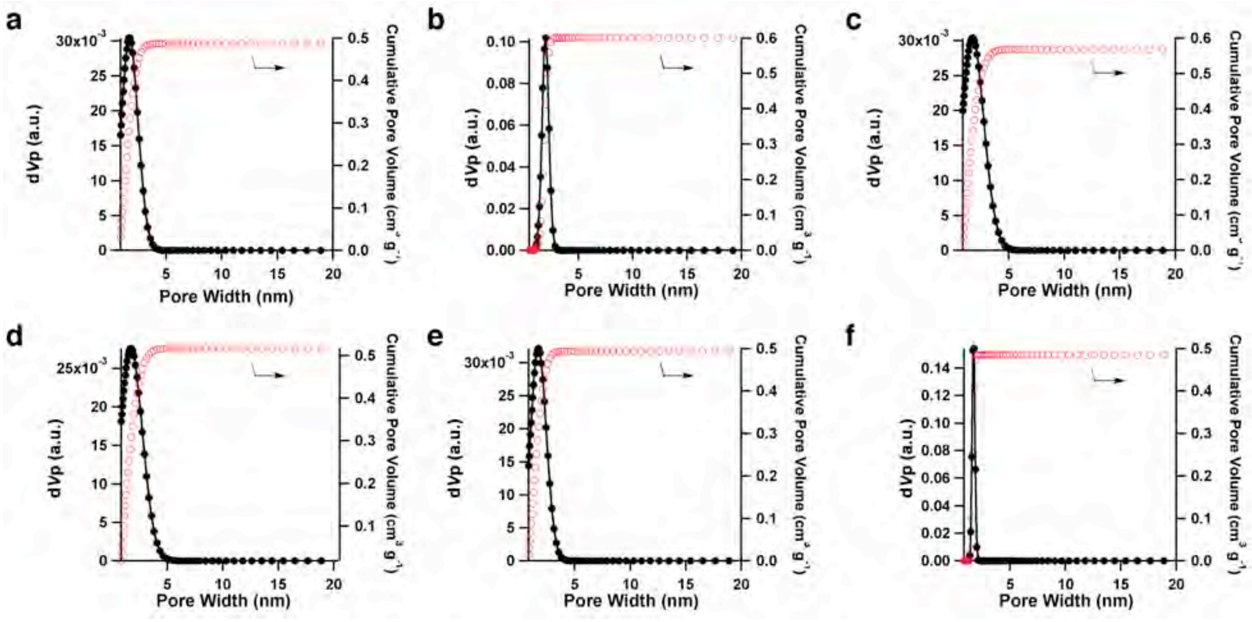

Supplementary Figure 5. Pore size and size distribution profiles of (**a**) H_2TPP-NiPc-COF, (**b**) H_2TPP-CuPc-COF, (**c**) ZnTPP-NiPc-COF, (**d**) ZnTPP-CuPc-COF, (**e**) CuTPP-NiPc-COF and (**f**) CuTPP-CuPc-COF.

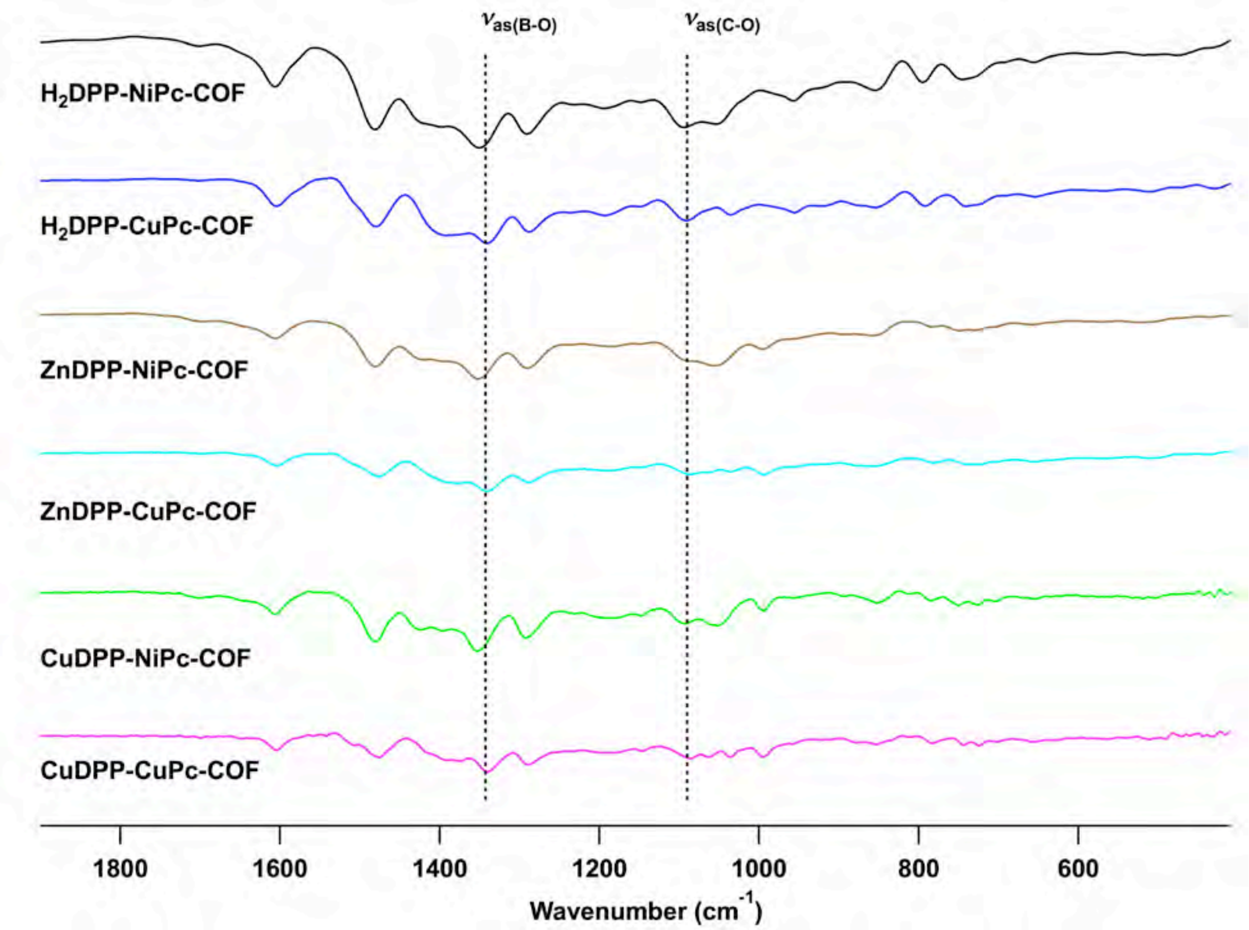

Supplementary Figure 6. IR spectra of H_2DPP-NiPc-COF, H_2DPP-CuPc-COF, ZnDPP-NiPc-COF, ZnDPP-CuPc-COF, CuDPP-NiPc-COF and CuDPP-CuPc-COF.

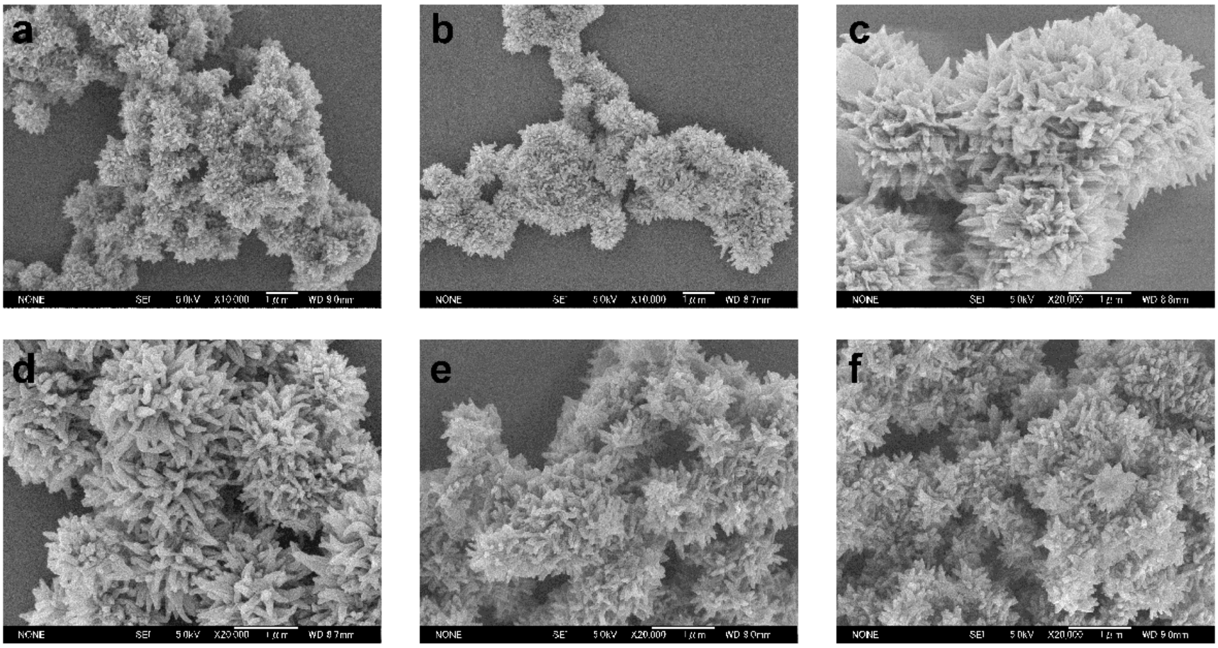

Supplementary Figure 7. FE-SEM images of (**a**) H_2DPP-NiPc-COF, (**b**) H_2DPP-CuPc-COF, (**c**) ZnDPP-NiPc-COF, (**d**) ZnDPP-CuPc-COF, (**e**) CuDPP-NiPc-COF and (**f**) CuDPP-CuPc-COF.

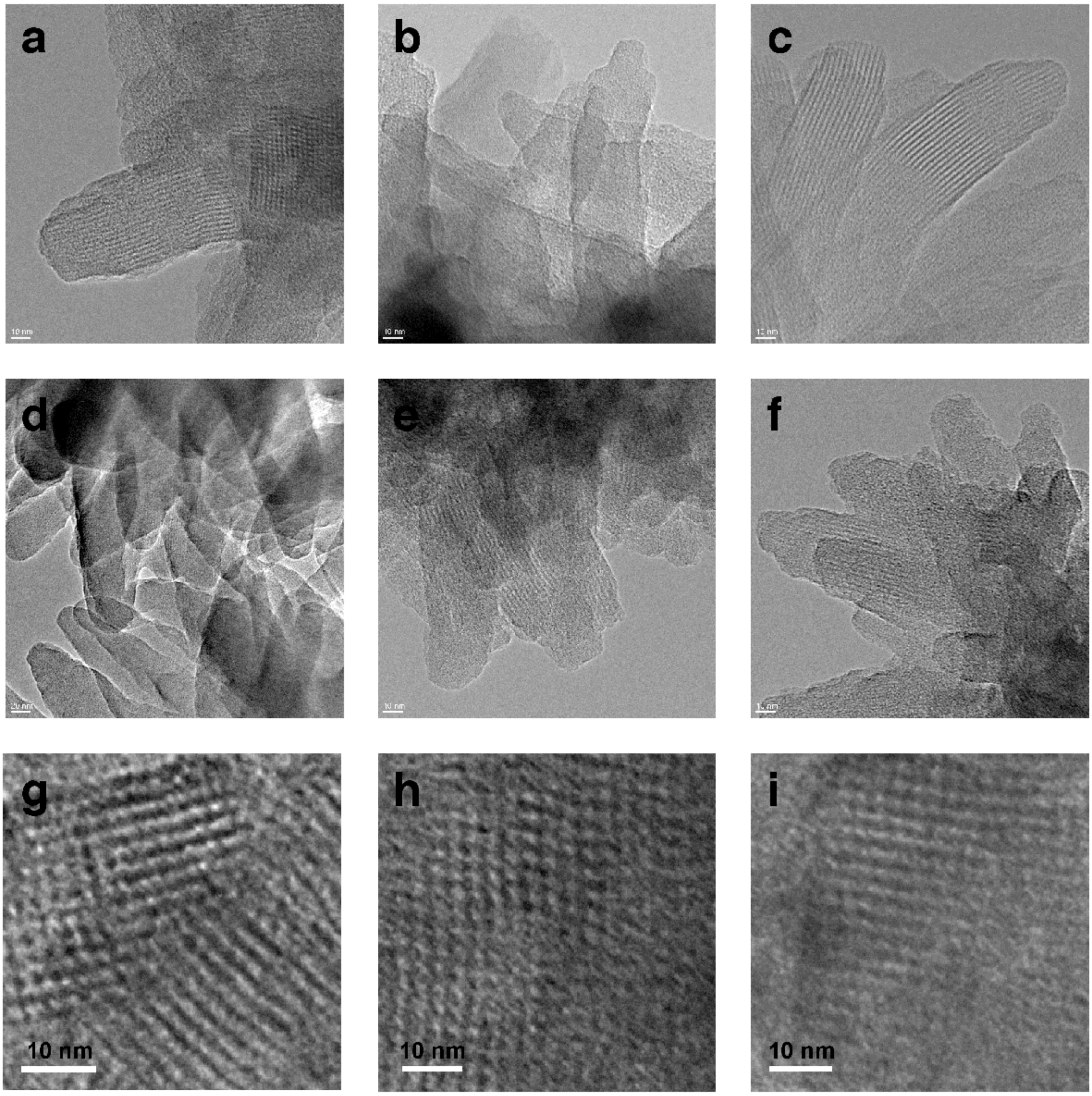

Supplementary Figure 8. HR-TEM images of (**a**) H_2DPP-NiPc-COF, (**b**) H_2DPP-CuPc-COF, (**c**) ZnDPP-NiPc-COF, (**d**) ZnDPP-CuPc-COF, (**e**) CuDPP-NiPc-COF and (**f**) CuDPP-CuPc-COF. (**g**)–(**i**) Enlarged HR-TEM image of (**g**) H_2DPP-NiPc-COF, (**h**) H_2DPP-CuPc-COF and (**i**) ZnDPP-NiPc-COF.

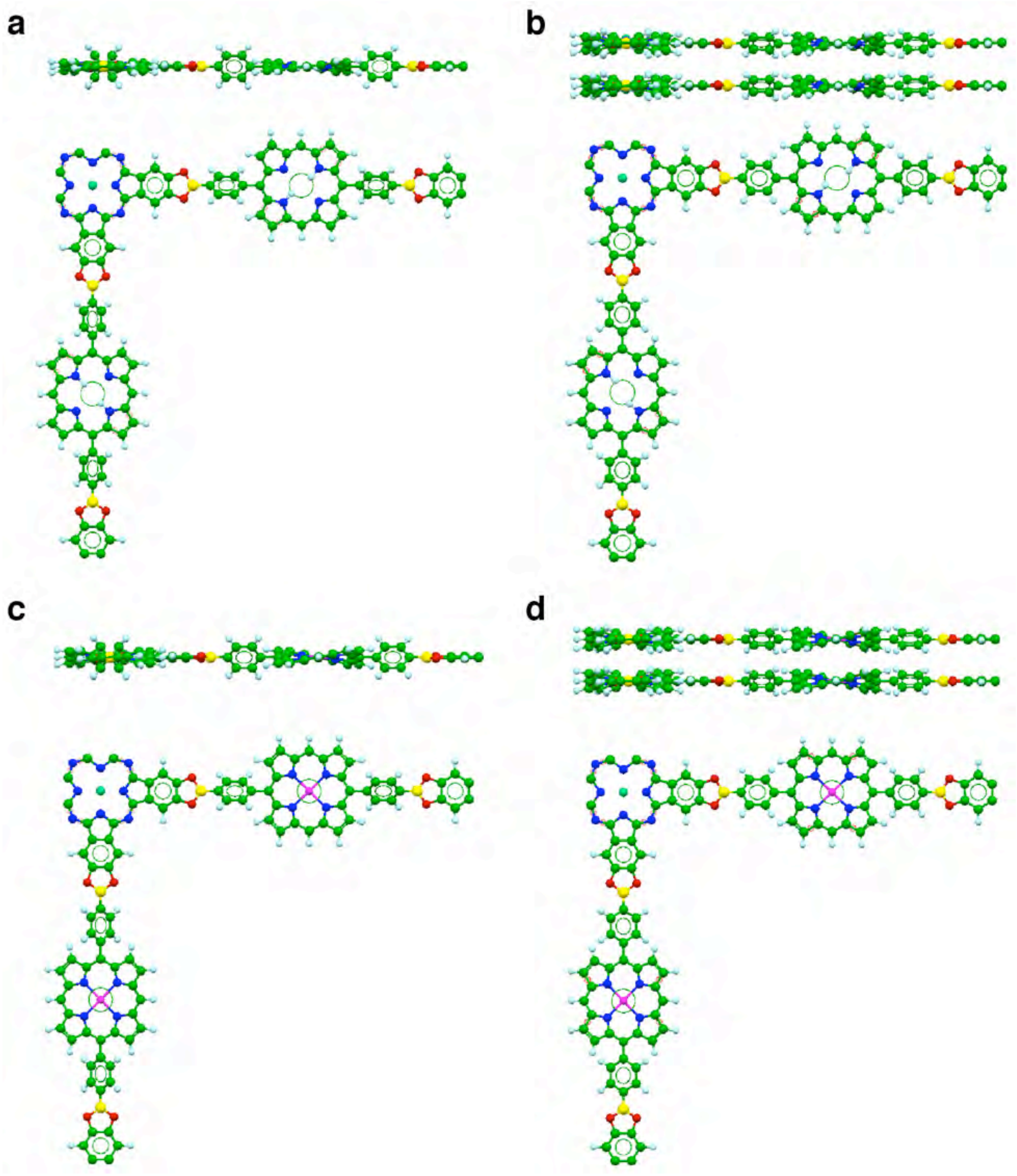

Supplementary Figure 9. (**a**) Optimized monolayer structure of H_2DPP-NiPc-COF along the *y* and *z* axes. (**b**) Optimized double-layer structure of H_2DPP-NiPc-COF along the *y* and *z* axes. (**c**) Optimized monolayer structure of ZnDPP-NiPc-COF along the *y* and *z* axes. (**d**) Optimized double-layer structure of ZnDPP-NiPc-COF along the *y* and *z* axes.

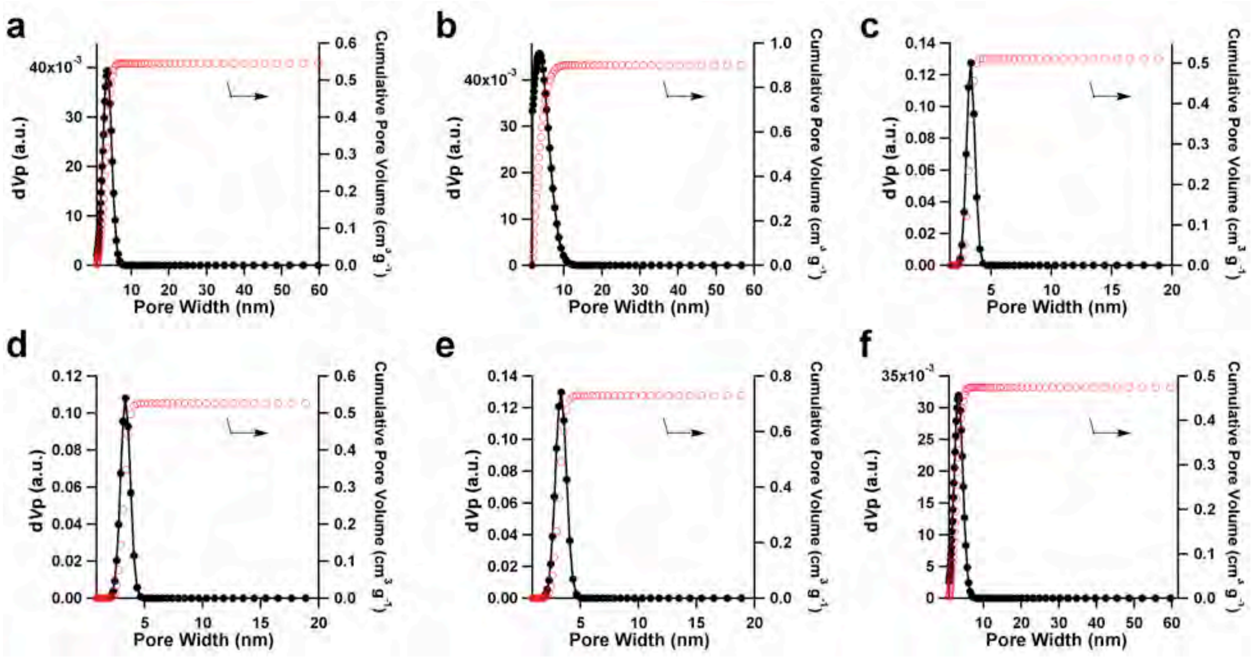

Supplementary Figure 10. Pore size and size distribution profiles of (**a**) H_2DPP-NiPc-COF, (**b**) H_2DPP-CuPc-COF, (**c**) ZnDPP-NiPc-COF, (**d**) ZnDPP-CuPc-COF, (**e**) CuDPP-NiPc-COF and (**f**) CuDPP-CuPc-COF.

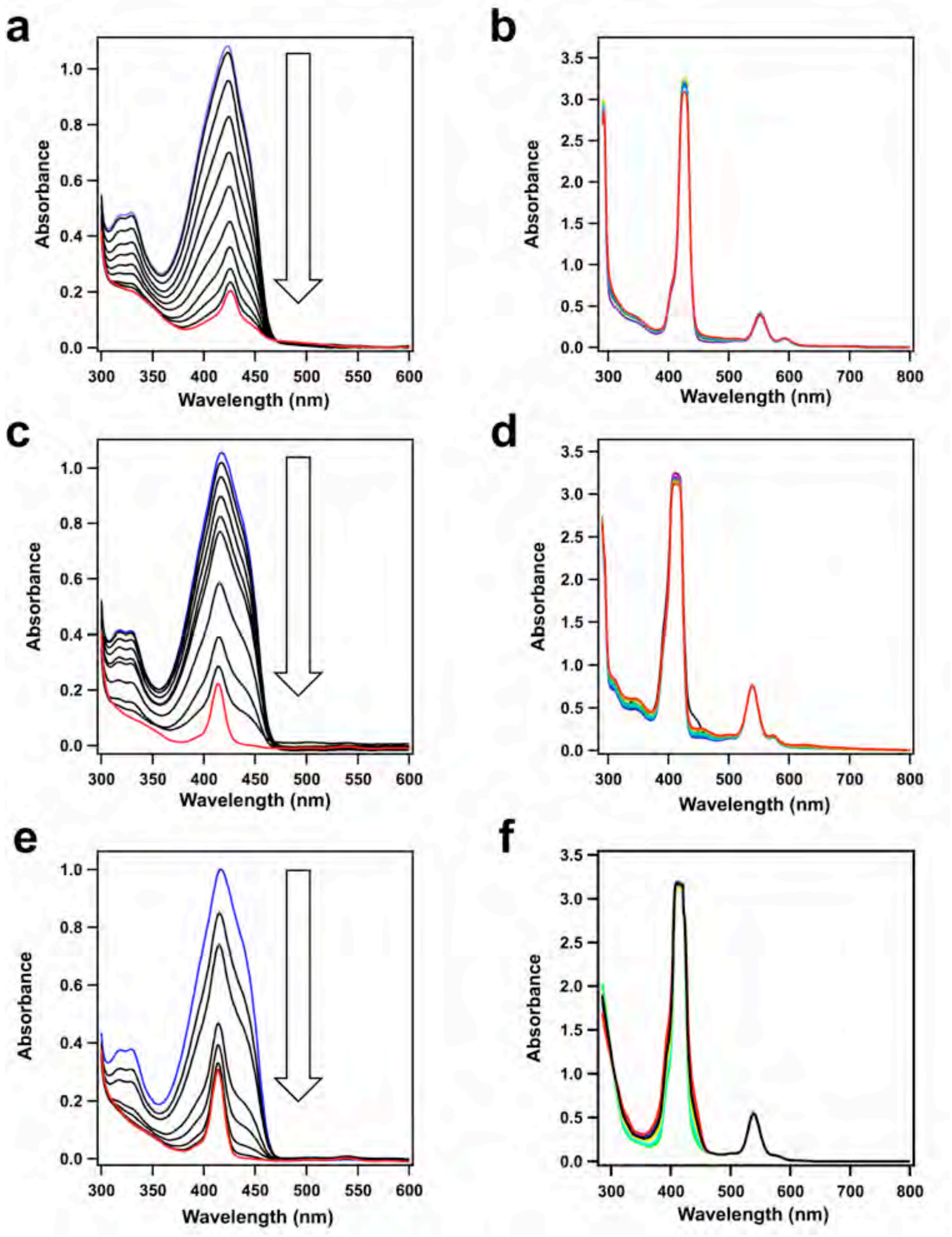

Supplementary Figure 11. Absorption spectral change of DPBF in the presence of (**a**) ZnTPP-CuPc-COF, (**b**) a mixture of ZnTPP and CuPc, (**c**) ZnDPP-CuPc-COF, (**d**) mixture of ZnDPP and CuPc, (**e**)ZnDPP-CuPc-COF, (**f**) mixture of ZnDPP and CuPc, in *o*-DCB. For **a**-**e**, the samples were irradiated at 500 nm. For **e** and **f**, the samples were irradiated at 750 nm.

Supplementary Materials and methods

Anhydrous *N*, *N*-dimethylacetamide (DMAc, 99.0%), *o*-dichlorobenzene (*o*-DCB), hexane, chloroform, KOH, $MgSO_4$, $Zn(OAc)_2{\cdot}2H_2O$, $Cu(OAc)_2{\cdot}H_2O$, Na_2SO_4 and $NaHCO_3$ were purchased from Kanto Chemicals. Methanol, anhydrous acetone (99.5%), toluene, HCl, triethylamine, 4-(1,3,2-dioxaborinan-2-yl)-benzaldehyde and anhydrous dichloromethane (99.0%) were purchased from Wako Chemicals. Mesitylene, tetrahydrofuran (THF), pyrrole, trifluoroacetic acid (TFA) and boron tribromide were purchased from TCI. Paraformaldehyde, boron trifluoride ethyl ether ($BF_3{\cdot}Et_2O$), *p*-chloranil and anhydrous *N*, *N*-dimethylformamide (DMF, 99.8%) were purchased from Aldrich.

^{1}H NMR spectra were recorded on JEOL models JNM-LA400 or JNM-LA500 NMR spectrometers, where chemical shifts (δ in ppm) were determined with a residual proton of the solvent as standard. Fourier transform Infrared (IR) spectra were recorded on a JASCO model FT-IR-6100 infrared spectrometer. UV-Vis-IR diffuse reflectance spectrum (Kubelka-Munk spectrum) was recorded on a JASCO model V-670 spectrometer equipped with integration sphere model IJN-727. The holder with solid samples of COFs and their controls in same amounts was mounted onto the window of the integration sphere. Matrix-assisted laser desorption ionization time-of-flight mass (MALDI-TOF MS) spectra were recorded on an Applied Biosystems BioSpectrometry model Voyager-DE-STR spectrometer in reflector or linear mode. Field-emission scanning electron microscopy (FE-SEM) was performed on a JEOL model JSM-6700 operating at an accelerating voltage of 5.0 kV. The sample was prepared by drop-casting an acetone suspension onto mica substrate and then coated with gold. High-resolution transmission electron microscopy (HR-TEM) images were obtained on a JEOL model JEM-3200 microscopy. The sample was prepared by drop-casting a sonicated acetone suspension of M_1TPP-M_2Pc-COFs and M_1DPP-M_2Pc-COFs onto a copper grid. X-ray diffraction (XRD) data were recorded on a Rigaku model RINT Ultima III diffractometer by depositing powder on glass substrate, from $2\theta = 1.5°$ up to 60° with 0.02° increment.

Nitrogen sorption isotherms were measured at 77 K with a Bel Japan Inc. model BELSORP-mini II analyzer. Before measurement, the samples were degassed in vacuum at 200 °C for more than 10 h. The Brunauer–Emmett–Teller (BET) method was utilized to calculate the specific surface areas. By using the non-local density functional theory (NLDFT) model, the pore size and size distribution were derived from the sorption curve.

We applied the density-functional tight-binding (DFTB) method to compute optimized

geometries and orbital energies of 2D porphyrin–phthalocyanine COFs. The calculations were carried out with the DFTB+ program package version 1.1[1]. DFTB is an approximate density functional theory method based on the tight binding approach, and utilizes an optimized minimal LCAO Slater-type all-valence basis set in combination with a two-center approximation for Hamiltonian matrix elements. The Coulombic interaction between partial atomic charges was determined using the self-consistent charge (SCC) formalism. Lennard–Jones type dispersion was employed in all calculations to describe van der Waals (vdW) and π-stacking interactions. Where possible, DFTB parameters from the dftb.org website[2] were employed as follows. Standard DFTB parameters for X–Y element pairs (X, Y = C, H, O, N) interactions were selected from the mio-0-1 set, B-H parameters were replaced by C-H from the same matsci-0-3 set to avoid the misleading bond between B and H atom. The results with replaced parameters were validated. B–X (X = C, O, N) related parameters were taken from the matsci-0-3 set, and Zn–X (X = C, H, O, N) parameters were taken from the znorg-0-1 set. Ni–X (X = C, H, O, N) parameters were taken from the trans3d-0-1 set. B-M (M = Zn, Ni) and Zn–Ni elements interactions are essentially negligible due to the large distance between metal and boron atoms. All calculations were carried out with fixed periodic boundary conditions (PBC). The single layer 2D model systems consisted of porphyrin and phthalocyanine units connected by bridges, which are kept the same orientations. The optimum lattice constants $a = b$ and c were determined by the lattice geometry optimizations.

Molecular modeling were conducted using Reflex, a software package for crystal determination from XRD patterns, implemented in MS modeling ver 4.4 (Accelrys Inc.)[3]. Simulated XRD patterns were calculated from the DFTB optimized AA and AB stacking structures.

As for the photocatalytic generation of singlet oxygen, an oxygen-saturated *o*-DCB solution (2.3 mL) of 1, 3-diphenylisobenzofuran (DPBF, 50 μM) in the presence of the COFs (0.5 mg) or controls (0.5 mg) in a quartz cell connected to an oxygen balloon was irradiated with lights at 500 or 750 nm (bandpass filter) from a Xenon lamp of Asahi Spectra model Max 301. The time-dependent electronic absorption spectra were recorded on a JASCO model V-670 spectrophotometer.

Synthetic procedures

$H_2TP_{BA}P$, $ZnTP_{BA}P$, $CuTP_{BA}P$, $NiPc[OH]_8$ and $CuPc[OH]_8$ were synthesized according to the methods in our previous reports[26, 28].

5,15-Di[4-(1,3,2-dioxaborinan-2-yl)phenyl]-21H,23H-porphine ($H_2DP_{BE}P$)

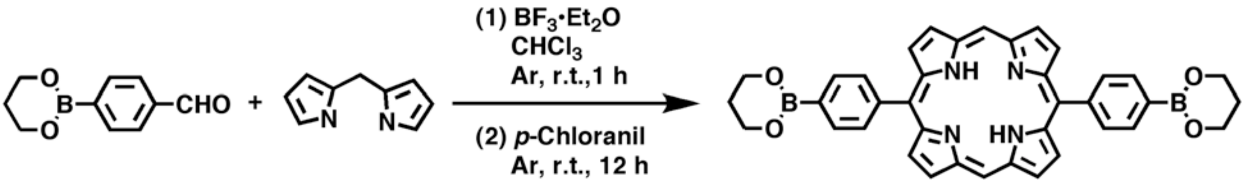

A chloroform solution (250 mL) containing 2,2'-dipyrrolemethane (0.3655 g, 2.5 mmol) and 4-(1,3,2-dioxaborinan-2-yl)-benzaldehyde (0.4750 g, 2.5 mmol) was degassed with a stream of Ar for 30 min. To the solution was added with $BF_3·Et_2O$ (78 μL). After the mixture was stirred at r. t. for 1 h under Ar, *p*-chloranil (1.48 g, 3.75 mmol) was added to the mixture, and the resulting mixture was further stirred for 12 h. The solvent was evaporated and the residue was purified by column chromatography (silica gel, $CHCl_3$) to give **$H_2DP_{BE}P$** in 23.8% yield. ^{1}H NMR (400 MHz, $CDCl_3$): δ (ppm) 10.40 (s, 2H, α-H), 9.45-9.46 (d, J = 4.6 Hz, 4H, β-H), 9.03-9.04 (d, J = 4.6 Hz, 4H, β-H), 8.19-8.27 (dd, J = 8.1 Hz, 8H, Ar-H), 4.30-4.33 (t, J = 5.5 Hz, 8H, CH_2), 2.19-2.22 (m, 4H, CH_2), -3.03 (s, 2H, NH of pyrrole). MALDI-TOF MS: *m*/*z* calcd 630.31 for $C_{38}H_{32}B_2N_4O_4$, found 630.15.

5, 15-Di[4-(dihydroxyboryl)phenyl]-*21H*,*23H*-porphine ($H_2DP_{BA}P$)

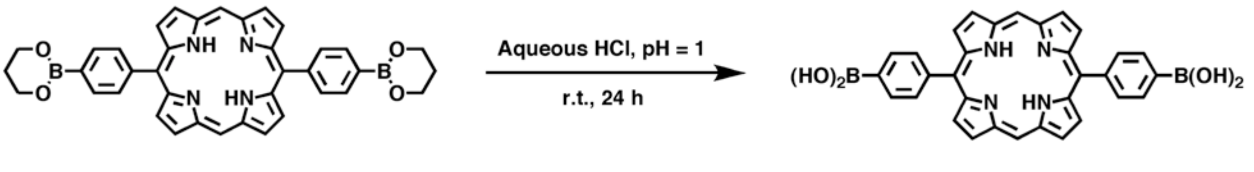

$H_2DP_{BE}P$ (100 mg) was dissolved in THF (100 mL), to which an aqueous solution of HCl (pH = 1.0, 50 mL) was added slowly. The mixture was stirred at room temperature for 24 h. CH_2Cl_2 (100 mL) was added to the mixture, and the organic layer was washed with aqueous solution of $NaHCO_3$ (100 mL × 3, 5 wt%) and water (100 mL × 3) and dried over anhydrous Na_2SO_4. The solution was concentrated under vacuum and poured into hexane (100 mL). The purple precipitate was collected by centrifugation and dried under vacuum to give **$H_2DP_{BA}P$** in 85% yield. ^{1}H NMR (400 MHz, d^8-THF): δ (ppm) 10.40 (s, 2H, α-H), 9.45-9.46 (d, J = 4.6 Hz,

4H, β-H), 9.06-9.07 (d, J = 4.6 Hz, 4H, β-H), 8.25-8.30 (dd, J = 1.2, 0.8 Hz, 8H, ArH), 7.51 (s, 4H, OH), -3.02 (s, 2H, NH of pyrrole). MALDI-TOF MS: *m*/*z* calcd 550.20 for $C_{32}H_{24}B_2N_4O_4$, found 550.32.

Zinc(II) 5, 15-di[4-(dihydroxyboryl)phenyl]porphine (ZnDP$_{BA}$P)

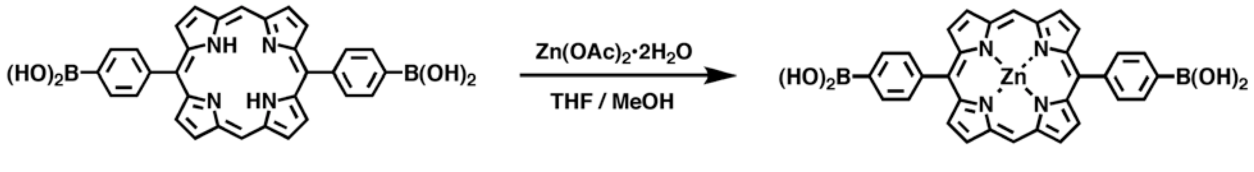

$H_2DP_{BA}P$ (110 mg, 0.2 mmol) was dissolved in THF (100 mL), to which a 100 mL methanol solution of $Zn(OAc)_2{\cdot}2H_2O$ (75 mg, 0.4 mmol) was added. The mixture was stirred at room temperature for 12 h. Water (100 mL) and CH_2Cl_2 (100 mL) was added to the reaction mixture, and the organic layer was collected and dried over anhydrous Na_2SO_4. The solution was concentrated and then poured into hexane. The red precipitate was collected by centrifugation and dried under vacuum to give **ZnDP$_{BA}$P** in 88% yield. ^{1}H NMR (400 MHz, d^8-THF): δ (ppm) 10.25 (s, 2H, α-H), 9.37-9.38 (d, J = 4.5 Hz, 4H, β-H), 9.02-9.03 (d, J = 4.5 Hz, 4H, β-H), 8.22 (d, J = 1.0, 1.0 Hz, 4H, ArH), 7.46 (s, 4H, OH). MALDI-TOF MS: *m*/*z* calc. 612.11 for $C_{32}H_{22}B_2N_4O_4Zn$, found 612.44.

Copper(II) 5, 15-di[4-(dihydroxyboryl)phenyl]porphine (CuDP$_{BA}$P)

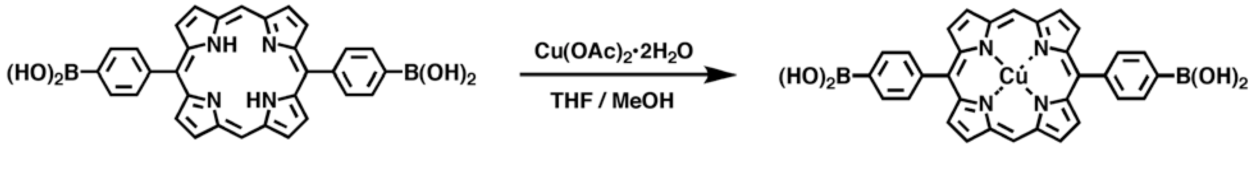

$H_2DP_{BE}P$ (110 mg, 0.2 mmol) was dissolved in THF (100 mL), to which a 100 mL methanol solution of $Cu(OAc)_2{\cdot}H_2O$ (42 mg, 0.25 mmol) was added. The mixture was stirred at room temperature for 30 min. Water (100 mL) and CH_2Cl_2 (100 mL) was added to the mixture, and the organic layer was collected and dried over anhydrous Na_2SO_4. The solution was concentrated and poured into hexane. The red precipitate was collected by centrifugation and dried under vacuum to give **CuDP$_{BA}$P** in 88% yield. MALDI-TOF MS: *m*/*z* calc. 611.11 for $C_{32}H_{22}B_2N_4O_4Cu$, found 611.32.

H_2TPP-CuPc-COF

A DMAc/*o*-DCB (2 mL, 1/1 by vol.) mixture of CuPc[OH]$_8$ (21.0 mg, 0.03 mmol) and $H_2TP_{BA}P$ (23.7 mg, 0.03 mmol) in Pyrex tube (10 mL) was degassed in by three freeze–pump–thaw cycles. The tube was sealed off and heated at 120 °C for 7 days. The precipitate was collected by centrifugation, washed with anhydrous DMAc and anhydrous acetone for 5 times, extracted by Soxhlet with anhydrous acetone for 3 days and dried at 150 °C under vacuum for 24 h, to give **H_2TPP-CuPc-COF** as a black solid in 78% isolation yield. Elemental Analysis: C (63.62), H (2.75), N (12.22); Cacl.: C (67.67), H (2.47), N (12.46).

ZnTPP-NiPc-COF

A DMAc/*o*-DCB (2 mL, 1/1 by vol.) mixture of NiPc[OH]$_8$ (21.0 mg, 0.03 mmol) and $ZnTP_{BA}P$ (25.6 mg, 0.03 mmol) in Pyrex tube (10 mL) was degassed by three freeze–pump–thaw cycles. The tube was sealed off and heated at 120 °C for 7 days. The precipitate was collected by centrifugation, washed with anhydrous DMAc and anhydrous acetone for 5 times, extracted by Soxhlet with anhydrous acetone for 3 days and dried at 150 °C under vacuum for 24 h, to give **ZnTPP-NiPc-COF** as a black solid in 75% isolation yield. Elemental Analysis: C (60.59), H (2.53), N (11.73); Cacl.: C (64.81), H (2.29), N (11.93).

ZnTPP-CuPc-COF

A DMAc/*o*-DCB (2 mL, 1/1 by vol.) mixture of CuPc[OH]$_8$ (21.0 mg, 0.03 mmol) and $ZnTP_{BA}P$ (25.6 mg, 0.03 mmol) in Pyrex tube (10 mL) was degassed by three freeze–pump–thaw cycles. The tube was sealed off and heated at 120 °C for 7 days. The precipitate was collected by centrifugation, washed with anhydrous DMAc and anhydrous acetone for 5 times, extracted by Soxhlet with anhydrous acetone for 3 days and dried at 150 °C under vacuum for 24 h, to give **ZnTPP-CuPc-COF** as a black solid in 79% isolation yield. Elemental Analysis: C (60.59), H (2.53), N (11.73); Cacl.: C (64.81), H (2.29), N (11.93).

CuTPP-NiPc-COF

A DMAc/*o*-DCB (2 mL, 1/1 by vol.) mixture of NiPc[OH]$_8$ (21.0 mg, 0.03 mmol) and $CuTP_{BA}P$

(25.5 mg, 0.03 mmol) in Pyrex tube (10 mL) was degassed by three freeze–pump–thaw cycles. The tube was sealed off and heated at 120 °C for 7 days. The precipitate was collected by centrifugation, washed with anhydrous DMAc and anhydrous acetone for 5 times, extracted by Soxhlet with anhydrous acetone for 3 days and dried at 150 °C under vacuum for 24 h, to give **CuTPP-NiPc-COF** as a black solid in 78% isolation yield. Elemental Analysis: C (61.86), H (2.41), N (11.78); Cacl.: C (64.89), H (2.29), N (11.95).

CuTPP-CuPc-COF

A DMAc/*o*-DCB (2 mL, 1/1 by vol.) mixture of CuPc[OH]$_8$ (21.0 mg, 0.03 mmol) and CuTP$_{BA}$P (25.5 mg, 0.03 mmol) in Pyrex tube (10 mL) was degassed by three freeze–pump–thaw cycles. The tube was sealed off and heated at 120 °C for 7 days. The precipitate was collected by centrifugation, washed with anhydrous DMAc and anhydrous acetone for 5 times, extracted by Soxhlet with anhydrous acetone for 3 days and dried at 150 °C under vacuum for 24 h, to give **CuTPP-CuPc-**COF as a black solid in 82% isolation yield. Elemental Analysis: C (62.67), H (2.38), N (11.72); Cacl.: C (64.67), H (2.29), N (11.91).

H$_2$DPP-NiPc-COF

A DMAc/*o*-DCB (2 mL, 2/1 by vol.) mixture of NiPc[OH]$_8$ (21.0 mg, 0.03 mmol) and H$_2$DP$_{BA}$P (33.1 mg, 0.06 mmol) in Pyrex tube (10 mL) was degassed by three freeze–pump–thaw cycles. The tube was sealed off and heated at 120 °C for 7 days. The precipitate was collected by centrifugation, washed with anhydrous DMAc and anhydrous acetone for 5 times, extracted by Soxhlet with anhydrous acetone for 3 days and dried at 150 °C under vacuum for 24 h, to give **H$_2$DPP-NiPc-COF** as a black solid in 75% isolation yield. Elemental analysis: C (64.68), H (3.18), N (13.38); Cacl.: C (69.65), H (2.92), N (13.54).

H$_2$DPP-CuPc-COF

A DMAc/*o*-dichlorobenzene (2 mL, 2/1 by vol.) mixture of CuPc[OH]$_8$ (21.0 mg, 0.03 mmol) and H$_2$DP$_{BA}$P (33.1 mg, 0.06 mmol) in Pyrex tube (10 mL) was degassed by three freeze–pump–thaw cycles. The tube was sealed off and heated at 120 °C for 7 days. The

precipitate was collected by centrifugation, washed with anhydrous DMAc and anhydrous acetone for 5 times, extracted by Soxhlet with anhydrous acetone for 3 days and dried at 150 °C under vacuum for 24 h, to give **H_2DPP-CuPc-COF** as a black solid in 79% isolation yield. Elemental Analysis: C (66.47), H (3.22), N (13.25); Cacl.: C (69.45), H (2.91), N (13.50).

ZnDPP-NiPc-COF

A DMAc/*o*-DCB (2 mL, 2/1 by vol.) mixture of NiPc[OH]$_8$ (21.0 mg, 0.03 mmol) and ZnDP$_{BA}$P (36.9 mg, 0.06 mmol) in Pyrex tube (10 mL) was degassed by three freeze–pump–thaw cycles. The tube was sealed off and heated at 120 °C for 7 days. The precipitate was collected by centrifugation, washed with anhydrous DMAc and anhydrous acetone for 5 times, extracted by Soxhlet with anhydrous acetone for 3 days and dried at 150 °C under vacuum for 24 h, to give **ZnDPP-NiPc-COF** as a black solid in 80% isolation yield. Elemental Analysis: C (63.68), H (2.72), N (12.89); Cacl.: C (67.16), H (2.58), N (13.05).

ZnDPP-CuPc-COF

A DMAc/*o*-DCB (2 mL, 2/1 by vol.) mixture of CuPc[OH]$_8$ (21.0 mg, 0.03 mmol) and ZnDP$_{BA}$P (36.9 mg, 0.06 mmol) in Pyrex tube (10 mL) was degassed by three freeze–pump–thaw cycles. The tube was sealed off and heated at 120 °C for 7 days. The precipitate was collected by centrifugation, washed with anhydrous DMAc and anhydrous acetone for 5 times, extracted by Soxhlet with anhydrous acetone for 3 days and dried at 150 °C under vacuum for 24 h, to give **ZnDPP-CuPc-COF** as a black solid in 78% isolation yield. Elemental Analysis: C (64.37), H (2.84), N (12.84); Cacl.: C (66.97), H (2.58), N (13.02).

CuDPP-NiPc-COF

A DMAc/*o*-DCB (2 mL, 2/1 by vol.) mixture of NiPc[OH]$_8$ (21.0 mg, 0.03 mmol) and CuDP$_{BA}$P (36.8 mg, 0.06 mmol) in Pyrex tube (10 mL) was degassed by three freeze–pump–thaw cycles. The tube was sealed off and heated at 120 °C for 7 days. The precipitate was collected by centrifugation, washed with anhydrous DMAc and anhydrous acetone for 5 times, extracted by Soxhlet with anhydrous acetone for 3 days and dried at 150 °C under vacuum for 24 h, to give

CuDPP-NiPc-COF as a black solid in 83% isolation yield. Elemental Analysis: C (65.46), H (2.72), N (13.01); Cacl.: C (67.23), H (2.59), N (13.07).

CuDPP-CuPc-COF

A DMAc/*o*-DCB (2 mL, 2/1 by vol.) mixture of CuPc$[OH]_8$ (21.0 mg, 0.03 mmol) and CuDP$_{BA}$P (36.8 mg, 0.06 mmol) in Pyrex tube (10 mL) was degassed by three freeze–pump–thaw cycles. The tube was sealed off and heated at 120 °C for 7 days. The precipitate was collected by centrifugation, washed with anhydrous DMAc and anhydrous acetone for 5 times, extracted by Soxhlet with anhydrous acetone for 3 days and dried at 150 °C under vacuum for 24 h, to give **CuDPP-CuPc-COF** as a black solid in 85% isolation yield. Elemental Analysis: C (64.67), H (2.69), N (12.96); Cacl.: C (67.04), H (2.58), N (13.03).

Supplementary References

1. B. Aradi, B. Hourahine, T. Frauenheim, *J. Phys. Chem. A* **111**, 5678 (2007).
2. http://www.dftb.org.
3. Accelrys, Material Studio Release Notes, Release 4.4, Accelrys Software, San Diego (2008).
